# Differential Thresholds of Proteasome Activation Reveal Two Separable Mechanisms of Sensory Organ Polarization in *C. elegans*

**DOI:** 10.3389/fcell.2021.619596

**Published:** 2021-02-09

**Authors:** Patricia Kunz, Christina Lehmann, Christian Pohl

**Affiliations:** Buchmann Institute for Molecular Life Sciences and Institute of Biochemistry II, Medical Faculty, Goethe University Frankfurt, Frankfurt, Germany

**Keywords:** sensory organ development, apical polarity, dendrite morphogenesis, proteasome, apical constriction, collective cell migration

## Abstract

Cephalization is a major innovation of animal evolution and implies a synchronization of nervous system, mouth, and foregut polarization to align alimentary tract and sensomotoric system for effective foraging. However, the underlying integration of morphogenetic programs is poorly understood. Here, we show that invagination of neuroectoderm through *de novo* polarization and apical constriction creates the mouth opening in the *Caenorhabditis elegans* embryo. Simultaneously, all 18 juxta-oral sensory organ dendritic tips become symmetrically positioned around the mouth: While the two bilaterally symmetric amphid sensilla endings are towed to the mouth opening, labial and cephalic sensilla become positioned independently. Dendrite towing is enabled by the pre-polarized sensory amphid pores intercalating into the leading edge of the anteriorly migrating epidermal sheet, while apical constriction-mediated cell–cell re-arrangements mediate positioning of all other sensory organs. These two processes can be separated by gradual inactivation of the 26S proteasome activator, RPN-6.1. Moreover, RPN-6.1 also shows a dose-dependent requirement for maintenance of coordinated apical polarization of other organs with apical lumen, the pharynx, and the intestine. Thus, our data unveil integration of morphogenetic programs during the coordination of alimentary tract and sensory organ formation and suggest that this process requires tight control of ubiquitin-dependent protein degradation.

## Introduction

Through sensory organs, animals can receive stimuli and transduce these to the nervous system to bring about a physiological change or behavioral response (Schmidt-Rhaesa, 2007). The most common form of sensory organs is cellular extensions such as microvilli and cilia, which harbor specific mechano-, chemo-, photo-, or thermoreceptors. Nematoda like Tardigrada and Euarthropoda use cuticular ciliary receptors. In the case of nematodes, these are either cuticular extensions (bristles/bristle-like structures) or cuticular pores. The nematode *Caenorhabditis elegans* exclusively uses cuticular pores where ciliated tips of sensory neuron dendrites either end in a cuticular channel that is connected to the exterior, project into a cuticular cavity, or end in a juxta-cuticular glial pocket (Altun and Hall, 2010).

The morphogenesis of *C. elegans* sensory organ dendrites is poorly understood. It has been initially suggested that “anterior neurons move toward the tip of the head, and the rudiments of the sensilla are formed; the neurons then move posteriorly again, sensory cell bodies laying down their dendritic processes as they go” (Sulston et al., 1983). This idea has been revisited a decade ago, and the morphogenetic mechanism of retrograde extension has been coined to describe this presumably particular mode of dendrite morphogenesis utilized here (Heiman and Shaham, 2009). In this mechanism, neurons tether their dendritic tips first anteriorly and then dendrites passively extend through the migration of posteriorly positioned neuron cell bodies in the opposite direction. It has been shown that a tight interaction between glia, their associated neurons and the surrounding epidermis is possible since both glia and neurons exhibit properties of epithelial cells that allow them to integrate into the surrounding epithelium (Low et al., 2019). Importantly, this mechanism was proposed specifically for the amphid sensilla, a bilateral pair of sensory organs both containing 12 sensory neuron dendrites (Altun and Hall, 2010). It has been suggested that although amphid sensilla dendrite morphogenesis requires anchoring of dendritic tips, the extension of dendrites is not driven by neuron migration but by towing of dendritic tips through the epidermis (Fan et al., 2019; Low et al., 2019). This is highly consistent with the timing of epidermal morphogenesis: The amphid sensory pore is embedded within the epidermis and connected to it through adherens junctions (Perkins et al., 1986). During embryonic elongation, which is driven by the epidermis, the amphid sensilla gain their specific elongated shape by establishing a connection to the epidermal sheet right before epidermal migration events occur (Fan et al., 2019). This connection is maintained during head enclosure (during which the epidermis encloses the anterior third of the embryo in a collective migration event) (Chisholm and Hardin, 2005). The connection between epidermis and dendritic tips requires DYF-7 (an extracellular protein required for anchoring), FRM-2 (a EPBL/moe/Yurt ortholog) (Low et al., 2019), SAX-7 (L1-type CAM), HMR-1 (E-cadherin), and DLG-1 (discs large) (Fan et al., 2019). In addition, it has been shown for arborized mechanosensory dendrites in *C. elegans* that the epidermis itself actively patterns dendritic morphogenesis, which, among other factors, also requires the cell adhesion molecule SAX-7 (reviewed in Yang and Chien, 2019). Taken together, the most recently proposed morphogenetic mechanism for amphid dendrites, dendrite towing, involves the neighboring tissue, the epidermis (Fan et al., 2019). This is in stark contrast to the retrograde extension mechanism proposed earlier (Heiman and Shaham, 2009). However, dendrite towing bears strong similarities to the morphogenetic mechanism described for a different sensory organ, the lateral line in zebrafish. Here, axonal growth cones co-migrate with their target cells, while their cell bodies remain stationary behind (Metcalfe, 1985). This has later been confirmed by time-lapse imaging and has been coined axon towing (Gilmour et al., 2004).

A presumably similar morphogenetic process to amphid sensilla formation has been previously described in *C. elegans*: In the male, the morphogenesis of a pair of prong-like sensory structures, called the copulatory spicules (Lints and Hall, 2009), is driven by the spicule socket cell. This socket cell guides the collective cellular movement that leads to the elongation of this sensory organ. The socket cell also creates the cuticular pore in which spicule dendrites end (Jiang and Sternberg, 1999). However, the relationship between socket cell migration and sensory dendrite elongation has not been addressed so far.

Notwithstanding a potential similarity of amphid to spicule dendrite morphogenesis, the mechanism of dendrite morphogenesis for the other 16 sensory organs of the head, the cephalic, and the inner and outer labial sensilla has not been addressed in any detail so far. It has been originally proposed that tissue folding might be involved: “a depression appears in the tip of the head; this does not involve morphogenetic cell death, and is presumably a way of providing more surface area for the sensilla” (Sulston et al., 1983). This suggests that a morphogenetic mechanism seems to be involved that can mediate tissue invagination. Tissue invagination during development often requires apical constriction as morphogenetic mechanism (Hunter and Fernandez-Gonzalez, 2017). Apical constriction can drive collective cellular re-arrangements through the polarized activation of actomyosin contractility, which has been characterized in *C. elegans* for gastrulation (Harrell and Goldstein, 2011; Pohl et al., 2012), ventral enclosure (Wernike et al., 2016), and pharynx morphogenesis (Rasmussen et al., 2012). The topological outcome of apical constriction is variable: It leads to scar-less cell internalization during gastrulation due to the lack of polarized junctions at this stage (Pohl et al., 2012), it induces cyst formation during pharynx morphogenesis (Rasmussen et al., 2012), and it often leads to the formation of tissue folds in other developing organisms (Hunter and Fernandez-Gonzalez, 2017).

During nervous system morphogenesis and dendrite morphogenesis in particular, substantial cellular remodeling has to occur. Such remodeling entails targeted degradation of proteins, protein complexes, and organelles, which can be brought about by the ubiquitin-proteasome system (UPS) or autophagy, respectively. It has been established that autophagy and the UPS are key contributors to neuron morphogenesis (Hamilton and Zito, 2013; Stavoe and Holzbaur, 2019). Inhibition of the proteasome, for instance, prevents neurite outgrowth (Laser et al., 2003) and proteasomes control axon and dendrite morphogenesis (Hamilton et al., 2012; Hsu et al., 2015). Moreover, the activity of the proteasome can be regulated by proteasome subunits that act as linking factors for the two main proteasomal subcomplexes, the 20S core and the 19S lid. It has become clear that the 19S component Rpn6/PSMD11 acts as a key regulator in this respect: Rpn6 stabilizes the otherwise weak interactions between the 19S lid and the alpha ATPase rings of the 20S proteolytic core (Pathare et al., 2012) and can be activated by stimulus-dependent phosphorylation (VerPlank et al., 2019). For instance, Rpn6-dependent regulation of proteasome activity has been documented for aging of the sub-ventricular zone (Wang et al., 2016). Looking beyond neuro-morphogenesis, Rpn6 seems to be generally involved in developmental decisions as it is required for efficient myofibroblast differentiation (Semren et al., 2015) and has to be down-regulated during drought stress in plants (Cho et al., 2015). Moreover, null alleles of *rpn6* in Drosophila (*l(2)k00103* and *rpn62F*) support embryo development; however, hatched larvae die at early stages, and it has been argued that this is due to an additive effect on cell proliferation rather than a distinct disruption of development (Lier and Paululat, 2002). This is similar in *C. elegans*, where RNAi of *rpn-6.1* has been shown to lead to substantial lethality with only 3% of embryos hatching (Takahashi et al., 2002). Importantly, it has been shown that increased levels of RPN-6.1 can protect animals from proteotoxic stress (Vilchez et al., 2012). However, the role of *rpn-6.1* in *C. elegans* morphogenesis has not been addressed yet.

Here, we investigate the mechanism of sensory organ dendrite morphogenesis during late stages of *C. elegans* embryogenesis. Specifically, we revisit observations originally made when determining the lineage of *C. elegans* (Sulston et al., 1983). We demonstrate that the mouth forms synchronously with overt morphogenesis of pharynx and nervous system. While the pharynx constricts, mouth and neuro-ectodermal cells invaginate at the anterior tip of the embryo through apical constriction. Remarkably, tissue invagination during mouth formation leads to movement of sensory organ endings on the surface of the head to their final positions at the tip of the embryo. In case of the amphid sensory organ, dendrite elongation occurs through the previously characterized mechanism of dendrite towing. However, unlike proposed earlier, sheath and socket glia cells seem to differentially contribute to towing. For all other head sensory organs, cell shape changes through *de novo* apical polarization, and subsequent apical constriction mediates their centripetal movement and symmetric placement of their juxta-oral, anterior endings. We show that these two processes not only occur through different morphogenetic mechanisms, we also uncover that they show a differential sensitivity to reduced proteasome activity. While amphid dendrite towing is highly sensitive to depletion of the key proteasome activator, RPN-6.1, apical constriction-dependent morphogenesis of all other sensory organs is much less sensitive. We reveal that other morphogenetic processes that are sensitive to RPN-6.1 depletion also involve *de novo* apical polarization. Hence, our results show that different morphogenetic mechanisms can couple epidermal morphogenesis to dendritic patterning and that these different mechanisms can be separated through titration of proteasome activity.

## Materials and Methods

### *Caenorhabditis elegans* Strain Maintenance

Strains used in this study were cultivated on NGM agar plates (Brenner, 1974) at 20–25°C feeding on OP50. Strain designations and genotypes are listed in Supplementary Materials.

### Mounting of Embryos

Embryos were mounted as described previously (Dutta et al., 2015), and time-lapse analysis was performed during the stages of early lima bean to 1.5-fold elongation. For imaging of head-on view embryos, we used Cellview cell culture dishes (Greiner Bio-One GmbH, Germany). All compartments of the dish were filled with M9. Embryos were arranged in one compartment, and 1 μl diluted 45.0 μm polystyrene microspheres (Polysciences, Inc., Warrington, PA, United States) were added. Embryos were positioned head-on through moving them with an eye lash within the M9 solution.

### Long-Term Imaging

Imaging was executed with a VisiScope spinning disk confocal microscope system (Visitron Systems, Puchheim, Germany). The system consists of a Leica DMI6000B inverted microscope, a Yokogawa CSU X1 scan head, and a Hamamatsu ImagEM EM-CCD. Z-sectioning was performed with a Piezo-driven motorized stage (Applied Scientific Instrumentation, Eugene, OR, United States) using a Leica HC PL APO 63X/1,4-0,6 oil objective. All acquisitions were performed at 20–23°C.

For most experiments, we collected z-stacks with 45 steps at 1.0 μm distance each with 2, 3, 4, or 5 min intervals, respectively, for a total duration of 1–3 h for acquiring early lima bean to 1.5-fold stage embryos. For imaging of the head-on-view, we used 3–4 min intervals to avoid tipping of embryos. For lineaging, we performed long-term imaging for 250 time points at 3 min intervals and with z sampling of 1 μm over a distance of 30 μm. For acquiring of embryos for one time point before and a time-lapse series after UV laser ablation, we used 2 min intervals and z sampling at 1 μm over a distance of 40–45 μm.

### UV Laser Ablation

For UV Laser ablation, we used a MLC03A-DPI VS-FRAP-control and VS-Laser Control system (Visitron Systems GmbH, Puchheim, Germany). For ablation of the AM pores, we positioned the UV laser at a PAR-6:GFP marked pore with 5 ms frap time per pixel with a target area diameter matching the diameter of the pore. Epidermal ablation was executed in proximity of the AM pore at a focal layer of ABDvab-10:mCherry marked epidermal tissue with 10 ms frap time per pixel in a wider diameter then for pore ablation. The laser was controlled manually by applying 3–8 frap cycles. All UV laser ablations were performed unilaterally, leaving the other side of the embryo intact as an internal control. We only took embryos into account, which were developing after laser ablation and where the morphogenesis of the AM dendrites on the non-ablated side was not affected.

### RNA Interference

We used the clone F57B9.10 from a commercially available library (Rual et al., 2004). On day 1 the clone was streaked on LB-ampicillin plates and cultured over night at 37°C. The next day a single clone was picked from the plate and cultivated over night at 37°C in LB-ampicillin. On day 3, the plasmid was extracted, and the sequence was verified through Sanger sequencing (using forward 5′-GTTTTCCCAGTCACGACGTT-3′ and reverse primer 5′-TGGATAACCGTATTACCGCC-3′). Afterward, the plasmid was amplified by PCR with a T7 primer (5′-TAATACGACTCACTATAGGG-3′). The PCR product was purified and transcribed into dsRNA (AmpliScribe T7 High Yield, Epicentre, Madison, WI, United States). Then, we diluted the dsRNA solution to the desired concentration in DEPC-treated M9 buffer.

For dsRNA injection, we prepared 2% agarose injection pads. These pads were dried at 37°C. Injection needles were pulled from borosilicate capillaries (Kwik-Fil 1B100F-4, World Precision Instruments, Sarasota, FL, United States) with a P-97 Flaming/Brown micropipette puller (Sutter Instruments, Novato, CA, United States) to obtain tapered, closed tips. After centrifugation, 1 μl of the dsRNA solution was filled into the injection needle. Afterward, the needle tip was broken open manually and the dsRNA solution injected into the ovaries of young adult *C. elegans*. For this, animals were positioned onto an agarose pad and immersed with a drop of halocarbon oil 700 (Sigma Aldrich, Steinheim, Germany). Injection of the dsRNA was accomplished with a Leica DMIL LED microscope with a 40×/0.75 PH2 air objective and Hoffman modulation contrast (Leica Microsystems, Wetzlar, Germany), a SMX micromanipulator (Sensapex Oy, Oulu, Finland), a MINJ-1 microinjector with a MINJ-4 needle holder (Tritech Research, Inc., Los Angeles, CA, United States), and an Einhell compressor (Einhell AG, Landau, Germany). For injection, we used 50–60 psi pressure and 1–8 injection cycles per animal. Animals were recovered in a drop of M9 on an OP50 seeded plate over night at 20°C. F1 offspring from injected animals was analyzed by imaging throughout the next day. We excluded embryos with obvious abnormalities, e.g., vacuoles, loss of marker signal, or extremely strong developmental defects. These embryos accounted for ∼30% of cases. The remaining ∼70% were analyzed by microscopy. Due to incomplete penetrance, we include a detailed quantification of phenotypes.

### Measurements and Lineaging

Movement of sensory organ pores was quantified in Fiji (ImageJ) (Schindelin et al., 2012) using a linear measuring tool. Specifically, we used maximum intensity *z*-stack projections of time lapse image series with a resolution of 7 pixels per μm. We measured movement length of pores or neurite tips every second or fifth time point relative to the position of the arcade cells’ apical anterior front, which becomes the most anterior, trackable part of the mouth. The length of the neurites was measured from the anterior border of their cell bodies to the tip of the neurites (directly adjacent to the AM pores). The position of AM cell bodies was analyzed through measuring the distance from the middle of the AM cell body assembly to the apical anterior front of arcade cells. All measurements were normalized to the length of each individual embryo. Tracking of arcade morphogenesis and lineaging of AM organ development was performed manually, by tracking apical surfaces and cell membranes or by tracking nuclei from four-cell stage embryos in time-lapse *z*-stacks and manual highlighting tracked structures in Fiji. Statistical analysis [two-way ANOVA and two-stage linear step-up procedure (Benjamnini, Kreiger, and Yekutieli)] was performed in GraphPad Prism 9 (for details of ANOVA statistics, see Supplementary Material).

## Results

### Amphid Pores Move Simultaneously With All Superficial Pores

**FIGURE 1:**
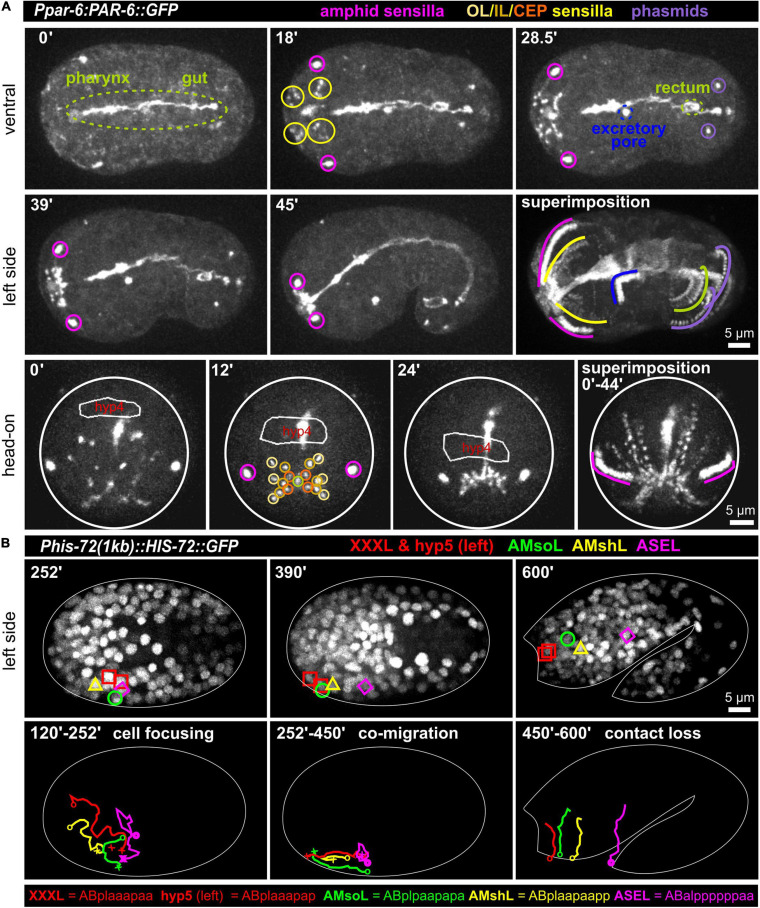
Simultaneous polarization and movement of apical pores in *C. elegans* during embryonic elongation. **(A)** Maximum intensity projections of stacks of confocal images from time lapse recordings of ventral (top), left side (middle), and head-on (bottom) views of wt embryos expressing PAR-6:GFP, highlighting the anterior directed movements of the amphid, outer labial (OL), inner labial (IL), cephalic (CEP), and phasmid sensilla pores during lima bean to 1.5-fold stage. See also Supplementary Video 1. PAR-6:GFP additionally highlights pharynx, gut, rectum, and the excretory pore. In the head-on view, the anterior-most hypodermal cell is traced (hyp4, traced based on a membrane marker which is not shown for clarity). **(B)** Maximum intensity projections of stacks of confocal images from time lapse recordings of lateral (left side) views of a representative wt embryo expressing GFP-marked histones from lima bean to twofold stage. Movement of the XXXL, hyp5 (hypodermal cell), AMsoL (amphid socket left), AMshL (amphid sheath left), and ASEL (amphid neuron cell body) are lineaged. Images in panel **(B)** required time-dependent brightness adjustment due to onset and increased expression of the histone from its zygotic promoter. See also Supplementary Video 2.

At the tip of the *C. elegans* head, a total of 18 epithelial sense organs, composed of ciliated dendritic endings of bipolar sensory neurons ensheathed by a single sheath (proximal) and one or more socket glia (at the distal end), can be found (Altun and Hall, 2010). They comprise sensilla, symmetrically positioned around the mouth: A bilateral pair of amphid (AM) sensilla, the four-fold symmetric cephalic (CEP), and the six-fold symmetric inner (IL) and outer labial sensilla (lateral outer labial, OLL; quadrant outer labial, OLQ, which are adjacent to the CEP sensilla). Since all these sensilla require an apical lumen for the dendrite endings of sensory neurons to integrate into or penetrate through the cuticle, we reasoned that apical polarity factors (aPARs, abnormal embryonic PARtitioning of cytoplasm) should highlight them. Accordingly, we found that PAR-3 (see below; using *it298[par-3:GFP]* and PAR-6 (using different markers: *xnIs3[par-6:PAR-6:GFP]*; *it319[par-6:GFP]*; *asIx1928[par-6:mCherry:PAR-6]*) (see also Supplementary Materials) not only highlight apical polarization of tubular organs (intestine, rectum, excretory pore) at this stage but also highlight all socket cells (pores) of these sensilla (Figure 1A and Supplementary Video 1). Remarkably, apical polarization of all structures that open on the surface of the embryo, including the mouth (see below), bilateral anterior deirid sensilla (data not shown), the excretory pore, the rectum, and the bilateral phasmid sensilla also occur simultaneously. Sheath cells do not seem to undergo apical polarization along their extensions that ensheath sensory neuron dendrites. The posterior deirid sensilla pores are not highlighted since they are only present at the L2 larval stage (Altun and Hall, 2010). At the same time, when the elongation of the embryo starts, the AM, IL, OLQ, OLL, and CEP sensilla pores move anteriorly to become symmetrically positioned around the prospective mouth (Figure 1A superimposition and Supplementary Video 1). The amphid pores start the movement from the most posterior-lateral position and follow the other pores at a distance to reach their final position, which is posterior-lateral relative to the other sensilla pores. Together with the pores, the anterior epidermal cell hyp4 also moves anteriorly (Figure 1A, bottom panel), demonstrating that sensilla pore movement occurs during head enclosure (see below).

### Sensory Organ Assembly Through Complex Cell Trajectories

The amphid sensillum is composed of the socket cell (AMso), the sheath cell (AMsh) and neuronal cell dendritic tips (of the ASE, ASG, ASH, ASI, ASJ, ASK, ADF, and ADL neurons). Its apical part has been shown to be completely embedded in the epidermis (and later in the cuticle). To better understand its developmental trajectory, we lineage traced AM sensillum cells (using the ASE neuron as an example for AM neurons) and the two adjacent anterior-most epidermal cells (XXX and hyp5, XXX later delaminates and forms a neuron-like cell; Altun and Hall, 2009a) during the early lima bean to the twofold stage of embryonic elongation (Figure 1B and Supplementary Video 2). We discovered complex trajectories that lead to focusing of all cells during mid-embryogenesis. After focusing, the epidermal cells hyp 5 and XXXL, AMso, and, at a small distance, AMsh move anteriorly. AMsh stops migrating already before the other cells do. Importantly, the ASE cell body does not move anteriorly with the other AM cells after focusing. Subsequently, the close contact between AM cells is lost, and, during elongation, cells adopt positions that resemble those known from larval/adult neuroanatomy. These observations show that the anterior movement of the amphid pores is coupled to the anterior migration of epidermal and socket glia cells while neuronal cells are stationary.

### Coupling of Epidermal Migration and AM Pore Movement

**FIGURE 2:**
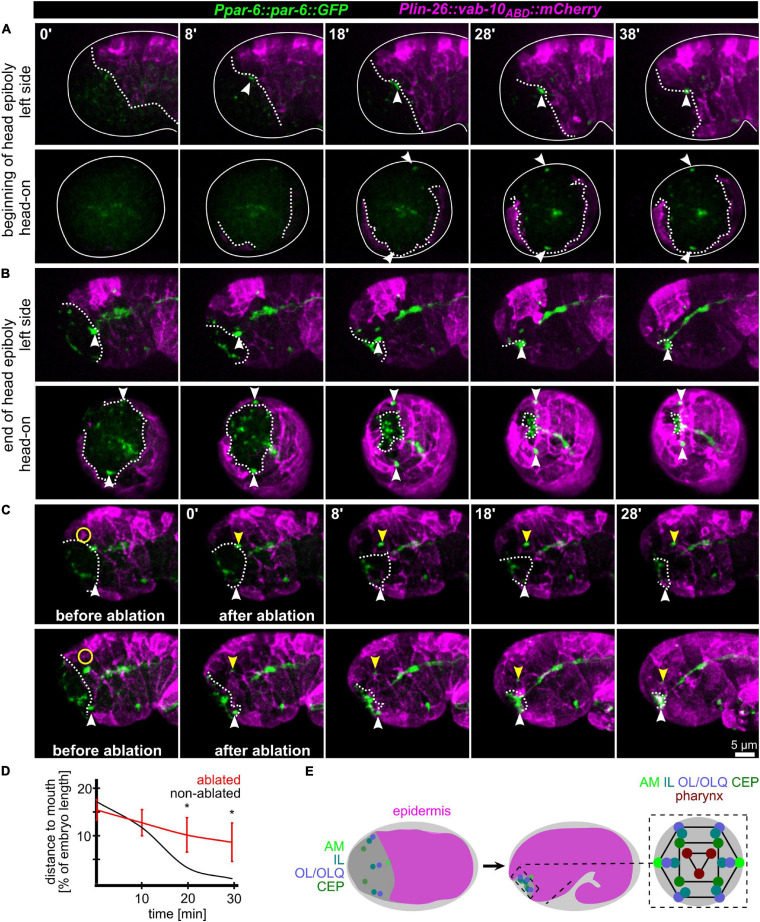
Epidermis migration and AM pore movement. **(A)** Maximum intensity projections of stacks of confocal images from time lapse recordings of left (top) and head-on (bottom) views of epidermis movement and movement of AM pores during lima bean stage. See also Supplementary Video 3. **(B)** Same as panel **(A)**, however, stage is lima bean to 1.5-fold stage. **(C)** Lateral view of embryos before and after UV laser ablation of the epidermis close to the AM pore. White dashed lines highlight the epidermal front. White arrowheads point to the position of the AM pore, and yellow arrowheads mark the position of the pore close to the ablated epidermis. Yellow circle marks the position of the laser ablation. See also Supplementary Video 4. **(D)** Quantification of epidermis ablation experiments (*n* = 4). The relative distance of the ablated (red) and un-ablated (black) AM pores to the mouth was measured. *P*-values from a multiple unpaired t-test and with two-stage linear step-up procedure (Benjamnini, Kreiger, and Yekutieli) are shown (*≥0.05; **≥0.01). **(E)** Left and middle: Schematic depicting the spreading of the epidermis around the head (head enclosure) and the initial and final positions of the sensory organ pores. Note that only the AM pores are located at the anterior epidermal edge. Right: Schematic depicting a juxta-oral cross section that highlights the symmetry of sensory organ pores and the symmetry of the pharynx.

Since AM sensilla are embedded in the epidermis, we wanted to clarify the role of head enclosure as part of the mechanism driving AM dendrite morphogenesis. Therefore, we investigated epidermal migration together with PAR-6, visualizing AM pores and the apical lumen of other pores and tubular organs. Consistent with what has been shown earlier (Fan et al., 2019; Low et al., 2019), at the beginning of the head enclosure, AM pores are forming laterally at the anterior epidermal front (Figure 2A, arrowheads and Supplementary Video 3). Together with progressive migration of the epidermal front, AM pores move anteriorly, finally reaching the prospective mouth at the end of epidermal enclosure (Figure 2B, arrowheads and Supplementary Video 3). To corroborate a physical link between epidermis and AM pores, we performed UV laser ablation of the epidermis directly adjacent to the AM pore (Figure 2C and Supplementary Video 4). We did so since our tracking analysis revealed that the epidermal hyp4 cell is among the first head epidermal cells to show marker expression from an epidermal promotor (*lin-26*; see also Supplementary Materials). Importantly, we designed our laser ablation experiments to not disturb epidermal migration and movement of the non-ablated AM pore on the other side of the embryo. By doing so, we found head enclosure by the epidermis to be uncoupled from AM pore movement. AM pore movement stops after ablation while epidermal migration is largely not affected (Figures 2C,D, yellow arrowheads and Supplementary Video 4). We conclude that a physical connection between the AM pore and the migrating epidermal tissue is essential for correct positioning of AM dendrite tips. However, our analysis also revealed that the movement of the remaining sensilla pores of the head is not directly coupled to the spreading of the epidermis over the head. Instead, these sensilla pores always form and move in front of the epidermal edge to obtain their final symmetric position (Figures 2B,E and Supplementary Video 3).

### Coupling of Epidermis Migration to AM Dendrite Elongation

**FIGURE 3:**
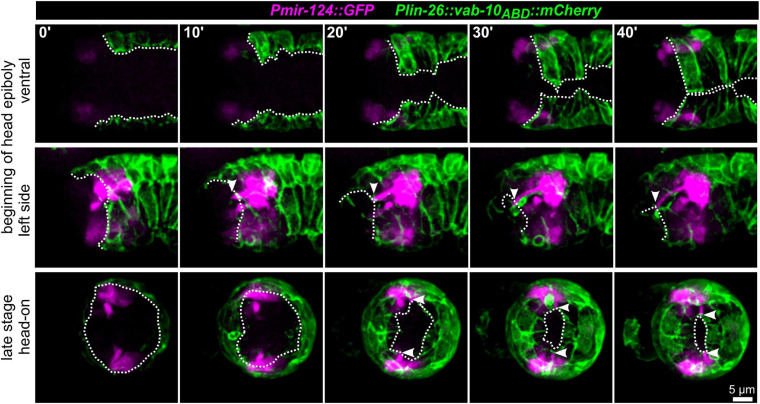
Epidermal enclosure and AM dendrite extension. Maximum intensity projections of stacks of confocal images from time lapse recordings of ventral (top), left side (middle), and head-on (bottom) view of wt embryos from lima-bean to 1.5-old stage. AM cell bodies and dendrites are highlighted in magenta and the epidermis in green. White arrowheads point to the position of AM dendrite tips at the anterior edge of the epidermis. White dashed lines mark the anterior and ventral edges of the epidermal sheet. See also Supplementary Video 5.

**FIGURE 4:**
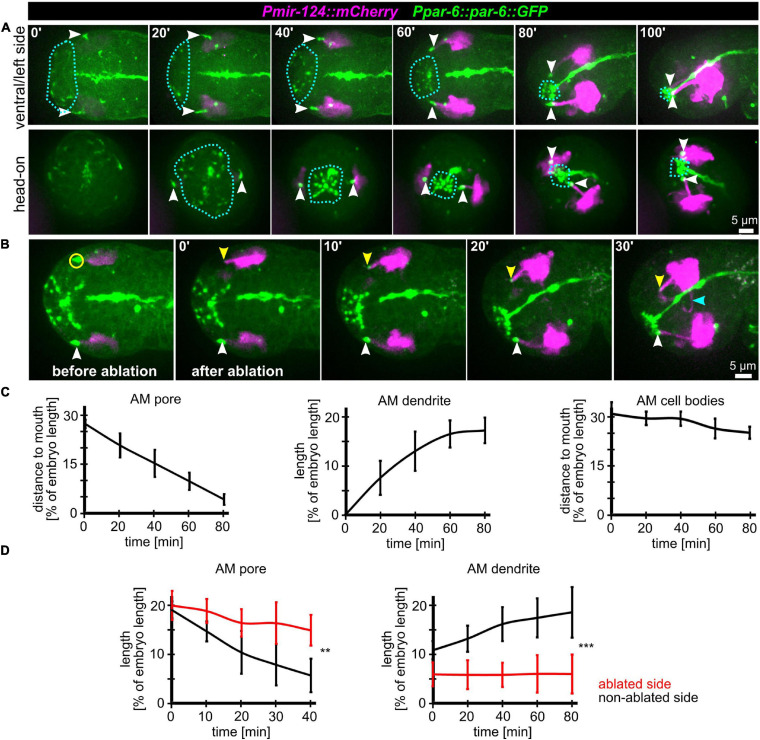
Coupling of AM pore movement to AM dendrite elongation. **(A)** Maximum intensity projections of stacks of confocal images from time lapse recordings of ventral/left side (top) and head-on (bottom) view of wt embryos from lima-bean to 1.5-old stage. AM cell bodies and dendrites are marked in magenta, and pores and apical lumen of pharynx and intestine are marked in green. Dashed lines indicate the anterior epidermal edge. White arrowheads mark the AM pores. See also Supplementary Video 6. **(B)** Depictions as in A; ventral/left side view of a representative embryo before and after UV laser-ablation of one of the two AM pores. The ablated area is marked by a yellow circle, the non-ablated AM pore is marked by a white arrowhead, and the ablated AM pore is marked by a yellow arrowhead. See also Supplementary Video 7. **(C)** Quantifications of the distance of the AM pore to the mouth (left), AM dendrite elongation (middle) and position of the AM neuron cell bodies relative to the mouth (right) (*n* = 5). **(D)** Quantifications of AM pore movement (left) and AM dendrite length in embryos where one AM pore was UV laser ablated, and the pore or dendrite on the other side was measured as internal control (*n* = 5; ±SD; two-way ANOVA *P*; ** ≥ 0.01; *** ≥ 0.001).

To further corroborate that AM dendritic tips move together with AM pores, we constructed strains that allowed us to monitor dendrite morphogenesis directly and together with epidermal sheet or pore movement. We found that microRNA promotor strains constructed previously (*mjIs27 [mir-124p:GFP* + *lin-15(*+)*]* and; *mjEx142 [mir-124p:mCherry];* Clark et al., 2010) are perfectly suited to monitor AM sensory organ dendrite morphogenesis. Specifically, this reporter is expressed in many AM neurons embedded in the AM pore (ASE, ASH, ASI, ASK) and AM neurons associated with the AM sheath (AWA, AWB, AWC). This reporter is also expressed in a limited set of other neurons with ciliated dendrites, an IL neuron (IL1) and – much weaker – phasmid neurons (PHA, PHB) (Supplementary Video 5). We found that expression of the reporter starts shortly after ciliated neurons are born, which is at the beginning of epidermal morphogenesis (ventral closure; Figure 3, top panel and Supplementary Video 6). Subsequently, when the anterior edge of the epidermis extends over the AM neuron cell bodies, dendrites and their sensory tips can be clearly discriminated (Figure 3, middle and bottom, arrowheads and Supplementary Video 6, white arrowheads). Consistent with the tracking data (Figure 1B and Supplementary Video 2) and the analysis of pore movement (Figure 2 and Supplementary Video 3), we observed a coupled movement of AM pores together with AM neuron dendritic tips (Figure 4A, arrowheads and Supplementary Video 5). While pores and dendritic tips move, neuron cell bodies stay stationary (Figure 4C, right panel). Thus, the movement of the pore seems directly coupled to dendrite elongation, which is also apparent from quantifications of pore movement and dendrite elongation (Figure 4C, left and middle panel). Moreover, in contrast to AM pore movement, the pores of all other sensory organs also move at that time (Figure 4A, dashed lines and Supplementary Video 6), however, they are always ahead of the epidermal border (Figure 2). To corroborate the coupling of epidermal migration and dendrite elongation, we again performed UV laser ablation, in this case, we ablated one of the AM pores. Ablation caused dendrite elongation arrest while the neurites on the control side elongated normally to the prospective mouth (Figures 4B,D and Supplementary Video 7). Notably, the remaining part of the pore and dendrites stayed associated and dendrites arrested during elongation; neurons of ablated AM organs even formed commissures (Figure 4B, blue arrowhead and Supplementary Video 7). This clarifies that the ablation of epidermis or AM pores does not destroy the whole sensory organ architecture but is specific for the targeted area and does not interfere with other aspects of its development.

Taken together, we conclude that head enclosure by the epidermis, AM pore movement and the elongation of AM dendrites are connected morphogenetic processes. We infer that AM dendrites are elongated trough the migration of the epidermis-attached AM pores while all AM neural cell bodies stay stationary. These findings are generally consistent with recent reports (Fan et al., 2019; Low et al., 2019) but are difficult to reconcile with AM dendrite morphogenesis through retrograde extension (Heiman and Shaham, 2009).

### Depletion of RPN-6 Disrupts AM Morphogenesis

It has been shown that components of the two main catabolic pathways, the UPS and autophagy, regulate transitions during *C. elegans* development (e.g., Zhang et al., 2009; Du et al., 2015). Especially, regulation of the oocyte-to-embryo transition is probably the best example for evolutionary conservation of UPS’ role (Verlhac et al., 2010). Nevertheless, besides the oocyte-to-embryo transition and a thorough analysis of embryonic differentiation programs (Du et al., 2015), little is known about the role of the UPS in *C. elegans* morphogenesis. This is most likely due to targeting of the UPS having been considered to lead to pleiotropic developmental phenotypes. Motivated by pioneering work on specific roles of basal UPS factors in Drosophila neuro-morphogenesis (reviewed in Hegde and Upadhya, 2007), we decided to test roles of the UPS in *C. elegans* sensory organ morphogenesis. By happenstance, we first characterized the role of *C. elegans’* ortholog of Rpn6/PSMD11 for AM morphogenesis. RPN-6.1 is an evolutionarily highly conserved component of the 26S proteasome lid, which is crucial to connect the core and regulatory proteasomal subcomplexes (Pathare et al., 2012) and hence has been coined proteasome activator.

**FIGURE 5:**
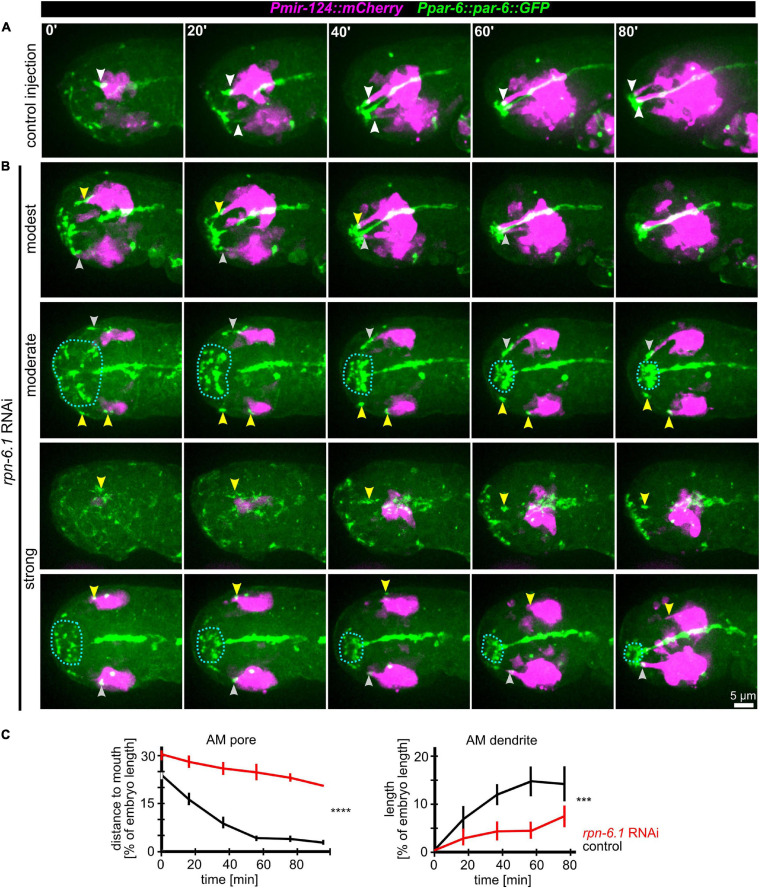
RPN-6.1 is required for proper AM pore and dendrite morphogenesis. **(A)** Maximum intensity projections of stacks of confocal images from time lapse recordings of left side views of embryo after control injection illustrating the movement of the sensilla pores (green) correlated with elongation of the AM dendrites (magenta). White arrowheads point to AM pores/dendrite tips. **(B)** Depictions as in A, however, *rpn-6.1* RNAi embryos are shown. Injection of dsRNA leads to different levels of penetrance (from top to bottom). Yellow arrowheads highlight impaired AM pore and/or dendrite morphogenesis. See also Supplementary Video 8. **(C)** Quantification of the effects of *rpn-6.1* RNAi on AM pore movement (left) and on AM dendrite morphogenesis (right) (*n* > 5, ±SD; two-way ANOVA *P*; *** ≥ 0.001; **** ≥ 0.0001).

Even moderate depletion of RPN-6.1 by RNAi preserved expression of all markers used in this study (including apical, neuronal, and epidermal) and allowed us to rule out pleiotropic effects such as general problems in cell fate acquisition (see below). The first obvious phenotypes under these conditions are failures in AM pore movement and dendrite elongation (Figures 5A,B and Supplementary Video 8). Importantly, irrespective of impaired AM pore movement, all other sensilla pores move to the prospective mouth like in wt (Figure 5B, middle and bottom panel and Supplementary Video 8). Specifically, the AM pore on one side moves normally and its dendrite fully extends, while the AM pore on the other side of the same embryo either becomes stretched, moves only partially, dendritic tips detach from the pore (in some cases only the anterior part of an AM pore reaches the prospective mouth subsequently), or an AM pore fails to be properly established and move (Figure 5B, arrowheads in top panels). These phenotypes are striking in several ways: First, they reveal that the first vulnerability of embryonic development after depletion of RPN-6.1 is the proper spatiotemporal coordination of AM sensory organ morphogenesis; second, they show that titration of RPN-6.1 levels can result in stochastic, unilateral phenotypes; third, cell fates seem to be by and large unaffected, for instance, the ciliated neuron-specific marker expressed from the *mir-124* promotor does not show any changes (Figure 5B). Strong phenotypes after RPN-6.1 depletion include total arrest of AM pores while the pores of other sensilla still reach the prospective mouth (Figure 5B, middle panels). Here, AM neurites are also not migrating in a thick bundle like in wt but often show only thin and fragile connections to the AM pore. Very strong depletion results in a complete lack of sensilla pore movement including AM, CEP, OLQ, and IL pores (Supplementary Video S12). Quantification of moderate cases of RPN-6.1 depletion shows a highly significant perturbation of AM pore movement and, consistently, also a strong reduction of AM dendrite bundle extension (Figure 5C).

We further investigated whether the specific role of RPN-6.1 in sensory organ morphogenesis is linked to epidermal sheet migration during head enclosure. To do so, we evaluated different degrees of RPN-6.1 depletion. In cases of modest and moderate depletion of RPN-6.1, the epidermis still migrates anteriorly to cover the embryonal head like in wt (Supplementary Figures 1A,B, top panels). Concomitantly, AM pores are stretched anteriorly (Supplementary Figure 1B, top panel). Often, AM pores split, the anterior part of the pore keeps moving to the prospective mouth and the posterior part arrests posteriorly (Supplementary Figure S1B, bottom panels).

Taken together, our analysis shows a graded response to RPN-6.1 depletion with AM sensory organ morphogenesis being affected, while epidermis and other sensory organs in the head are not affected after strong depletion.

### RPN-6.1 Is Required for Proper Lumen Morphogenesis of the Alimentary System

**FIGURE 6:**
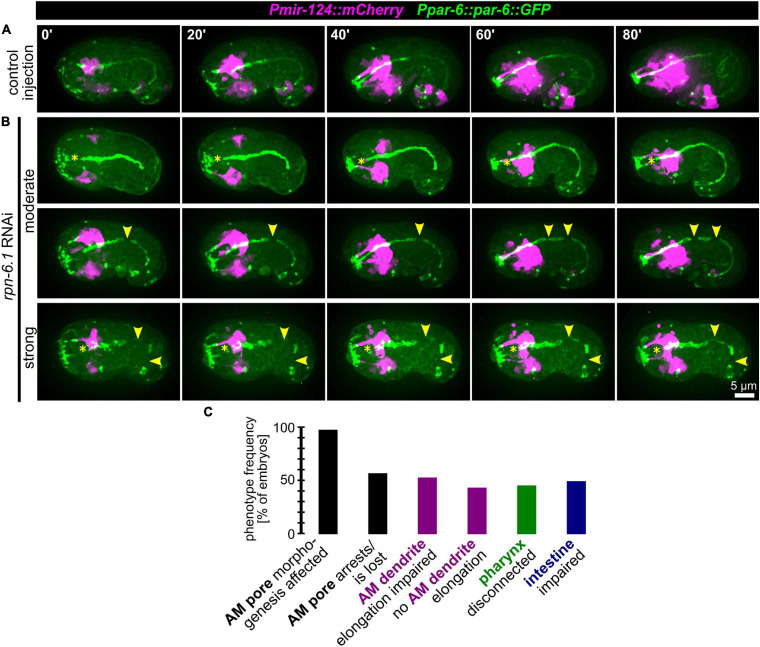
RPN-6.1 is required for the morphogenesis of organs with apical lumen. **(A)** Maximum intensity projections of stacks of confocal images from time lapse recordings of left side view of embryo after control injection; apically polarized surfaces (sensory organ pores, excretory pore, pharynx, intestine) are shown in green, and AM neurons and dendrites are shown in magenta. **(B)** Depictions as in A, however, for *rpn-6.1* RNAi embryos. Yellow arrowheads mark discontinuities of pharynx or intestine, respectively. See also Supplementary Video 9. **(C)** Quantification of phenotypes observed in *rpn-6.1* RNAi embryos (*n* = 29 for AM and *n* = 22 for all other phenotypes). None of the phenotypes has been observed in control injected embryos (*n* ≥ 20).

Given the gradual phenotypes for AM pore movement from stretching to splitting and arrest, we reasoned that the morphogenetic role of RPN-6.1 seems to lie in the coordination of inter- or intra-epithelial cell-cell contact formation for cells undergoing apical polarization. Consistently, when we analyzed the remaining structures in the embryo that are polarizing at this stage of development (using the apical polarity factor PAR-6 as a marker), we observed that, in addition to AM pore-specific defects, the pharynx often lacks a connection to the mouth (Figures 6A,B and Supplementary Video 9). Additionally, in many of the embryos where pharynx and mouth are not properly connected, the intestine is often discontinuous (Figure 6B). Concordantly, in *rpn-6.1* RNAi embryos with alimentary system phenotypes, pharynx and intestine show a lack of aligned apical surfaces and frayed lumens. Similar to the lack of a connection of the pharynx with the mouth, the intestine is often not connected to the rectal cavity (Figure 6B, bottom panels, yellow arrowheads).

From this data we conclude that RPN-6.1 plays a very specific, dose-dependent role for the correct spatio-temporal coordination of apical polarization and cell-cell attachment. AM sensilla morphogenesis constitutes the process most susceptible to RPN-6.1 depletion, followed by pharynx-arcade attachment, intestinal epithelization, and intestine-rectum attachment (to itself and to the rectum) (Figure 6C and Supplementary Figure 2).

### Coupling of Apical Constriction to Sensilla Pore Positioning

**FIGURE 7:**
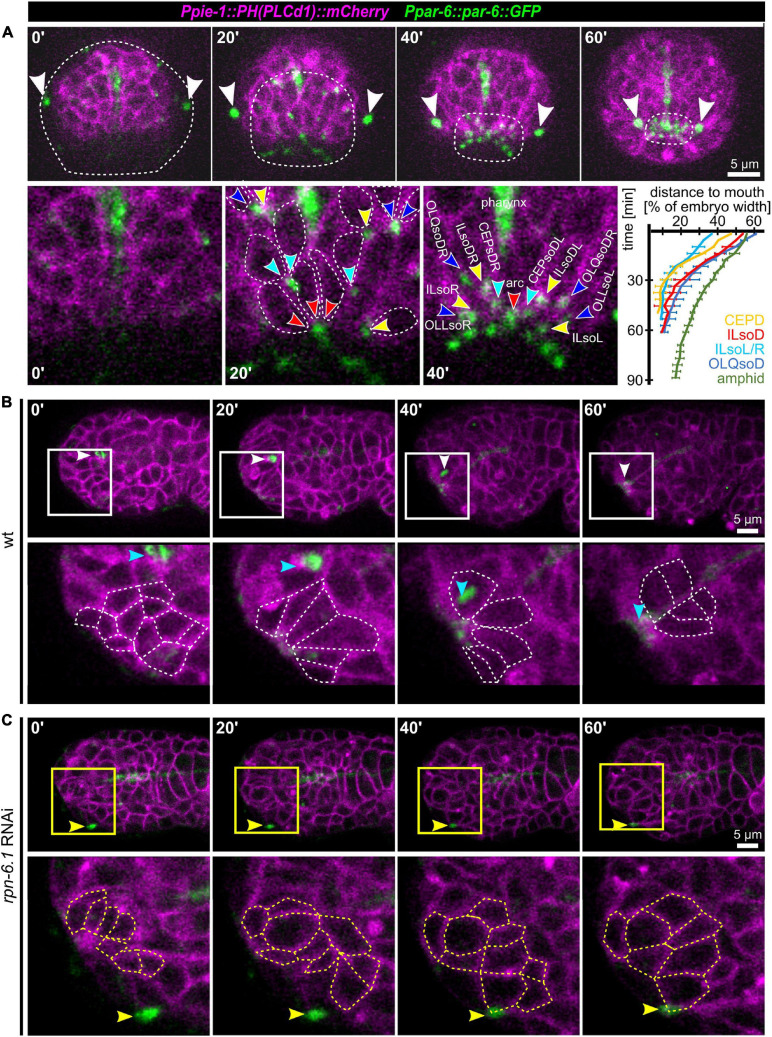
Apical constriction, sensory organ pore movement and the role of RPN-6.1. **(A)** Top: Maximum intensity projections of stacks of confocal images from time lapse recordings of a representative wt embryo imaged from anterior. The edge of the epidermis was tracked based on cell membranes and is highlighted by a dashed line. The AM pores are highlighted by white arrowheads. Bottom left: Magnified views of cell shape changes and identification of individual sensory organ socket cells. Anterior apical tips of cells are marked with arrowheads. Bottom right: Quantification of sensory organ pore (socket cell) movement (*n* = 5). **(B)** Top: Maximum intensity projections of stacks of confocal images from time lapse recordings of a representative wt embryo imaged from the left side. Bottom: Magnified single *z*-stacks from the boxed area above. Arrowheads mark the AM pore, individual neuronal and arcade cells are outlined to illustrate their shape change. **(C)** Representation as in panel **(B)**, however, for a representative, moderately RPN-6.1 depleted embryo. See also Supplementary Video 12.

After observing the simultaneous anterior movement of the head sensilla pores to the prospective mouth (Figure 1A), we analyzed OL, IL, and CEP sensilla pore movement relative to AM pore movement and epidermis migration in more detail. For marking the sensilla pores, we used PAR-6:GFP (*xnIs3[par-6:PAR-6:GFP* + *unc-119(*+)]), and for marking cell membranes, we used the phosphatidylinositol 4,5-bisphosphate-binding probe mCherry:PH(PLCdelta1) (Audhya et al., 2005). This combination enables simultaneous tracking of sensory organ pores and the anterior boundary of the epidermis (Figures 7A,B). In doing so, we found that the AM socket cells (AMsoL/R) move a distance of approximately ≥ 50% of embryo width (corresponding to 12–15 μm), a distance similar to that for the dorsal OLQ socket cells (OLQsoDL/R), followed by ILsoD (∼50%), dorsal CEP (∼50%), and the ILsoL/R (∼40%, corresponding to 10–12 μm). The movement distance and symmetry are due to the position of socket cells at the time of apical polarization (Figure 7A, bottom panels).

Taking an even closer look at pore movement shows that AM pores are kinematically separable from all other pores: While CEP, IL, and OLQ sensilla pores reach their juxta-oral position simultaneously, AM sensilla pores show a marked delay (Figure 7A, bottom right). Moreover, as indicated above, all sensilla pores but the AM pores are anterior to the edge of the migrating epidermal sheet that extends anteriorly to cover the head (Figure 7A, top panel). This shows that all head sensilla pores and dendrites except the AM pores seem to move by a mechanism different from towing through the epidermis.

Since pore migration is occurring at the same time as the formation of what has been called anterior sensory depression (Sulston et al., 1983), we conjectured that either apical constriction or basal expansion of cells must be involved. Only these two morphogenetic mechanisms have been shown to drive similar tissue dynamics. Hence, we further investigated the occurrence of cell shape changes at the anterior tip of the embryo. Within most cells that become positioned directly adjacent to the prospective mouth, we detected the formation of bottle-like shapes with an anterior accumulation of apical markers (Figure 7B and Supplementary Video 10). To confirm that apical constriction is indeed a main driver of sensory organ morphogenesis, we analyzed several other factors known to be involved in apical constriction (Supplementary Figure 3, Supplementary Video 11). We observed that actin (using lifeact as a probe) is transiently enriched at the anterior tip together with the non-muscle myosin II heavy chain (NMY-2; Supplementary Figure 3A). Actin is subsequently lost, while NMY-2 marks all sensory organ pores and the mouth (Supplementary Figure 3A, right panel). As shown previously, tubulin bundles that move toward the anterior tip of the head are apparent that represent sensory organ dendrites stabilized by the kinetochore-microtubule coupling machinery (Supplementary Figure 3B, dashed lines, arrowheads; Cheerambathur et al., 2019). In addition, apical constriction is also immediately apparent when analyzing the non-muscle myosin regulatory light chain (MLC-4), which stains sensory organ pores and other apically constricting cells from the point of their *de novo* apical polarization until late embryogenesis (Supplementary Figure 3C).

Since the cells that undergo apical constriction were not polarized before, we analyzed the sequence of events during *de novo* apical polarization. Consistent with apical constriction as mechanism underlying anterior tissue remodeling, the non-muscle myosin regulatory light chain (MLC-4, Supplementary Figure 3C) and NMY-2 (Supplementary Figure 3D) stain sensory organ pores (arrowheads) and other apically constricting cells (dashed lines) from the point of their *de novo* apical polarization until late embryogenesis. Moreover, examination of a strain co-expressing NMY-2 and PAR-6 showed that NMY-2 seems to localize to the nascent apical surface first, followed by the polarity determinant (Supplementary Figure 3D). Moreover, besides PAR-6 also PAR-3 shows *de novo* apical localization, suggesting that the whole aPAR complex becomes apically enriched at this stage (Supplementary Figure 3E).

The above data strongly suggest that collective apical constriction seems to mediate pore movement and mouth formation. We next tested whether RPN-6.1 affects collective apical constriction. As shown above, the degree of RPN-6.1 depletion allows us to separate AM pore and dendrite morphogenesis (modest/moderate depletion) from the morphogenesis of all other head sensory organs (strong depletion) (Figure 5B). When analyzing cases of strong RPN-6.1 depletion, we found that apical polarization of most cells at the anterior tip of the embryo is defective (Figure 7C), which results in a loss of collective cell behaviors (Supplementary Video 12). Therefore, we conclude that apical constriction within the anterior-most cells seems to constitute an important force which drives cellular re-arrangements that determine head sensilla pore positioning. We observed strong impairment of pore movement and loss of directed cell-shape changes within the anterior-most cells after RPN-6.1 depletion. Thus, RPN-6.1 is affecting correct apical constriction and thereby sensory organ placement.

### Interplay of *de novo* Juxta-Oral Apical Polarization and Sensory Organ Placement

**FIGURE 8:**
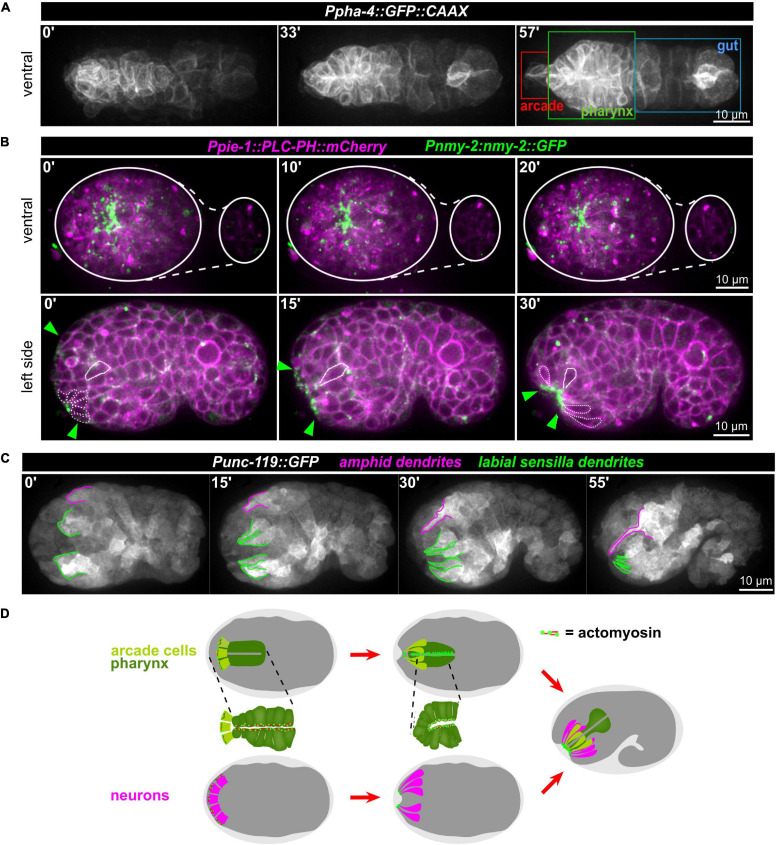
Apical constriction of anterior cells. **(A)** Maximum intensity projections of stacks of confocal images from time lapse recordings of a representative wt embryo expressing a membrane-localized GFP under the control of the *pha-4* promotor imaged from the ventral side. The individual compartments of the alimentary system are highlighted in the right panel. See also Supplementary Video 13. **(B)** Top: Maximum intensity projections of stacks of confocal images from time lapse recordings of representative wt embryo imaged from the ventral side. White lines mark the outlines of the embryo. Bottom: Central confocal plane from time lapse recordings of a representative wt embryo imaged from the left side. Green arrowheads mark the anterior cell surfaces that acquire and accumulate NMY-2. The dashed white line highlights cells that have undergone apical constriction. The solid white line traces a cell that undergoes apical constriction and thereby moves juxta-orally. See also Supplementary Video 10. **(C)** Maximum intensity projections of stacks of confocal images from time lapse recordings of a representative wt embryo imaged from the left side. Labial sensilla (green) and the left amphid sensillum (magenta) are tracked. **(D)** Schematic illustrating how sequential, collective apical constriction at the anterior tip of the embryo creates the anterior-most epithelial part of the mouth (arcade cells, light green) and, simultaneously, invagination of anteriorly localized neurons.

Anatomically, the mouth opening in *C. elegans* consists of three concentric rings of epidermal cells (from outside to inside: hyp3, hyp2, and hyp1, also called lips after deposition of cuticle) that are linked to the pharyngeal epithelium through two sequential rings of partially fused interstitial cells, the anterior and posterior arcade rings (consisting of three arcade cells in case of the anterior—arc ant DL, arc ant DR, and arc ant V—and six cells in case of the posterior ring—arc post D, arc post DL, and arc post DR, arc post V, arc post VL, and arc post VR) (Altun and Hall, 2009b). From previous work (Sulston et al., 1983) and anatomical descriptions based on EM analyses (Figure 8A, inset; Altun and Hall, 2009b), we reasoned that AM sensilla morphogenesis and formation of the connection between mouth and pharynx might be topologically and kinematically similar: An epithelial pore forms anteriorly to the pharynx primordium (Figure 8A). This pore consists of arcade cells, which resemble the socket glia of AM sensory organs. The pore connects to the pharynx lumen and opens to the outside by connecting to the anterior epidermis (Figure 8A). Accordingly, using a marker that highlights the cell membranes of all cells of the alimentary system (*Ppha-1:GFP:CAAX*), including the arcade cells, we observed apical polarization (Supplementary Video 13) and constriction (Figure 8B) of arcade cells. Furthermore, we find that apical polarization of arcade cells is not transient but persists until formation of a connection with the anterior, juxta-oral cells and the pharynx has been formed (Supplementary Video 13). Concomitant with apical constriction of arcade cells, numerous anteriorly located cells undergo apical constriction (Figure 8B, bottom panels and Figure 8C). Constriction first leads to the formation of local clusters that eventually integrate into a broad zone of apically constricted cells (Figure 8B, bottom panels). Many of these apically constricting cells are ciliated neurons of sensory cilia (Figure 8C) and their socket glia cells (Figure 7A). Apical constriction of these cells leads to an invagination of the embryo’s anterior tip and the formation of what has been coined anterior neuropore. However, unlike proposed earlier (Sulston et al., 1983), this neuropore does not evert, but stays in place until connected to the arcade epithelium (Supplementary Video 10).

These observations combined with seminal work on the embryonic lineage (Sulston et al., 1983), allow us to propose a working model for mouth and sensory organ morphogenesis (Figure 8D and Supplementary Figure 4). (1) Collective apical constriction drives invagination of the anterior tip of the embryo, where most sensory organ socket glia and neurons of the IL, OL, and CEP sensory organs are located (Supplementary Figure 4). (2) Simultaneously with apical constriction, the anterior spreading of the epidermis and the integration of the AM sensory organ into its migrating edge allow the AM dendrites to extend toward the other sensory organs and the mouth, all of which later forms the lips of the animal (Supplementary Figure 4, middle panel). (3) Unlike proposed earlier but fully consistent with lineage tracing (Sulston et al., 1983), the cell bodies of the AM sensory organ’s neurons stay where they were born and do not migrate (Supplementary Figure 4, bottom panel). (4) Through apical constriction and subsequent delamination, many cells that are initially located at the anterior tip reach their final positions, which are more posterior (e.g., non-sensory neurons like AVA, AVE, AVD, and RME). (5) Apical constriction triggers formation of a shallow funnel that allows the inner-most apically constricting cells, the arcade cells, to become surrounded by sensory organ dendrite tips.

Together, this means that three concurrent collective cellular behaviors mediate head morphogenesis: Tissue invagination by apical constriction, epidermal expansion linked to dendrite towing and delamination of inter- and motor-neurons. Our experiments show that apical constriction-mediated and epidermal spreading-mediated processes can be separated genetically and that the latter strongly depends on proteasome activation.

## Discussion

**FIGURE 9:**
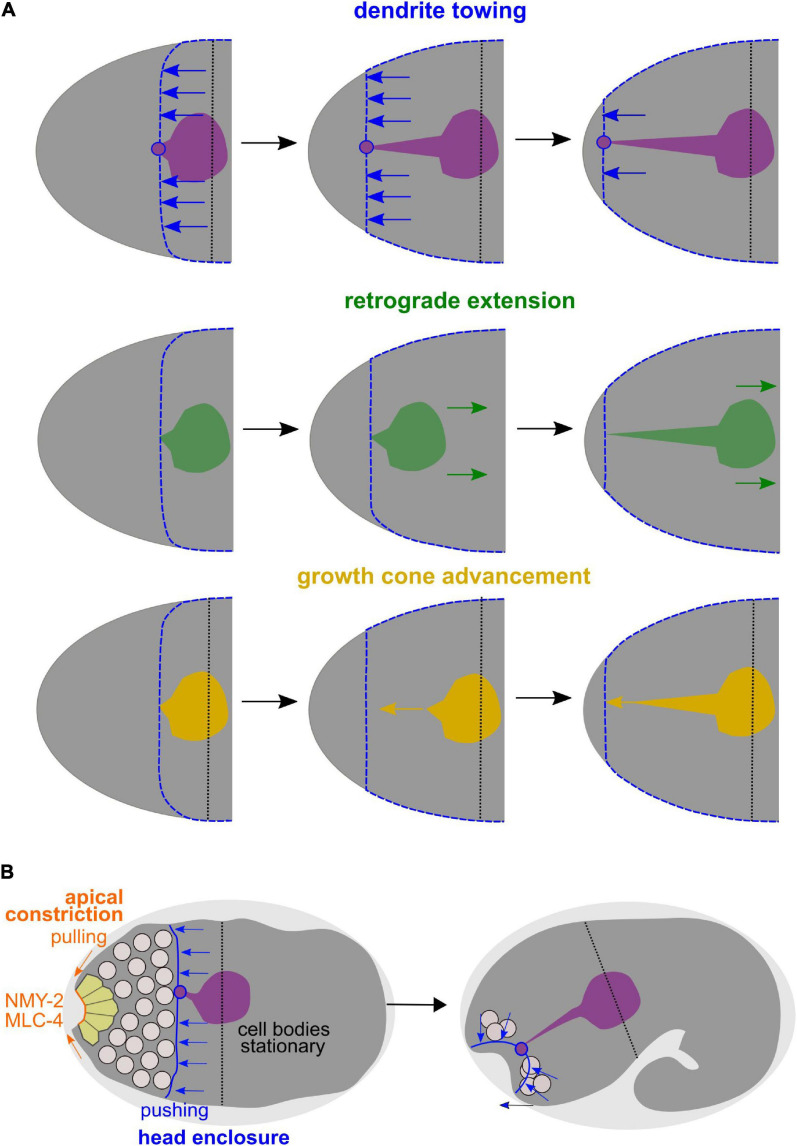
Dendrite towing governs AM dendrite morphogenesis and apical constriction contributes to placement of sensory organ pores. **(A)** Alternative models for AM dendrite morphogenesis. Top: During dendrite towing, the anterior-directed migration of the epidermis generates pulling forces that lead to movement of the AM sensory organ. Pulling forces can be transduced since the amphid organ partially intercalates into the epidermal layer through junctions of the socket glia cell, to which the dendrite tips of the AM neurons form contacts. Middle: In the case of retrograde extension, it has been claimed that the posterior-directed migration of neurons would lead to dendrite extension. Bottom: During growth cone advancement, dendrites would extend actively by forming a migratory specialization at their anterior ends. **(B)** Working model for collective morphogenesis of AM dendrites, neuron internalization, and mouth formation. Through dendrite towing, the anteriorly migrating epidermal sheet mediates AM sensory organ morphogenesis, while apical constriction of anteriorly located cells leads to tissue bending and formation of an anterior neuropore that connects to the mouth formed through apical constriction of arcade cells.

In the canonical case of neurite extension, a growth cone actively advances, thereby elongating the neurite (Figure 9A, bottom). While neurites use this mode of morphogenesis in *C. elegans* (Hedgecock et al., 1990), it has been claimed that AM sensory dendrites in the *C. elegans* embryo extend by anchoring of dendritic tips and migration of their cell bodies, a newly discovered mode of dendrite morphogenesis that has been coined retrograde extension (Heiman and Shaham, 2009; Figure 9A, middle). In contrast, our analyses and recently published work (Fan et al., 2019) show that AM sensilla extend by dendrite towing (Figure 9A, top). As already traced by Sulston et al. (1983), the neurons of the AM sensilla do not actively move during the time of dendrite extension. They also only passively acquire more posterior positions later, when many cells do so due to continued embryonic elongation. Neuronal cells get re-positioned when the pharynx extends, during which many of these cells can slide along its basement membrane. Thus, even this late and apparently posterior-directed change of neuron cell positions relative to the mouth cannot be considered retrograde extension but a passive movement. Passive movement of cell bodies is also reflected by the fact that many of these cells have variable positions in the adult organism. For instance, strong position variability has been well documented around the anterior bulb of the pharynx, for instance, for the OLQ socket glia (OLQsoDL/R; White et al., 1986; Altun and Hall, 2011).

Importantly, the difference between the morphogenesis of AM and all other head sensilla was already discussed much earlier in general anatomical and ontological descriptions of nematodes: “The bilaterally symmetrical amphids are separately innervated and cannot be considered a part of the cephalic papillary symmetry. Unlike the papillary nerves, the amphidial nerves enter the nerve ring indirectly, through a commissure and their original position probably was posterior to the labial region as indicated by embryonic rhabditis and adult phasmidians” (Chitwood and Chitwood, 1937). Thus, our data and the seminal work on the *C. elegans* lineage fully confirm this statement: AM sensilla are assembled posterior to all other head sense organs and are the only sensilla that directly form a union with the epidermis (Fan et al., 2019).

In addition, our analyses uncover that towing of amphid dendrites is accompanied by another morphogenetic process, the formation of the alimentary system and of all other sensory organs in the head by apical constriction (Figure 9B). Specifically, our analyses show that, spatio-temporally, coordinated apical constriction is involved in placing the dendritic endings of the head sensory organ endings except for the AM. Therefore, we propose that besides dendrite towing, apical constriction is the second main morphogenetic mechanism shaping sensory dendrites in *C. elegans*. Previously, it was shown that during gastrulation, *C. elegans* repeatedly utilizes a morphogenetic module that has been coined apical constriction initially (Lee and Goldstein, 2003). This module consists of internalizing cells which use centripetal, contractile actomyosin flow that they couple to cell-cell adhesion complexes of neighboring cells, thereby mediating their own covering (Roh-Johnson et al., 2012). However, during gastrulation, internalizing cells do not substantially deform but the covering cells do (Pohl et al., 2012). In contrast, during sensory organ morphogenesis, we now demonstrate that most cells at the anterior tip of the *C. elegans* embryo utilize *bona fide* apical constriction: (1) Cells deform and acquire a bottle-like morphology, (2) apical constriction leads to formation of a tissue indentation, which is a prototypic topological change in apical constriction (Pasakarnis et al., 2016), and (3) cells maintain high apical levels of non-muscle myosin II and of other apical polarity determinants and do not lose them like during gastrulation (Pohl et al., 2012). The latter aspect is probably best explained by the fact that the anterior endings of cells acquire and maintain an apical identity while cells lose their apical surface when gastrulating. Notably, it has been previously suggested that rosette formation is a main driver of morphogenesis for sensory organs (Fan et al., 2019). However, rosettes only represent a kinematic description of local cell positions in a planar configuration and do not take the three-dimensional cell shape into account. Our data show that the main transient configuration is bottle-shaped cells, which appear like rosettes when focusing on their apical aspect only.

Moreover, our data reveal that differential thresholds of proteasome activation allow to separate two morphogenetic mechanisms, AM dendrite towing by the epidermis and apical constriction-mediated assembly of all other sensory organs. Thereby, our data on the main proteasome activator, RPN-6.1, directly contribute to the growing list of specific functions of proteasome subunits in *C. elegans* development: Specific functions in sex determination have been shown for RPN-10 (Shimada et al., 2006), spindle rotation during early embryogenesis depends on the subunit RPN-2 (Sugiyama et al., 2008), or insulin/IGF-1 signaling requires the proteasome-associated de-ubiquitylating enzyme UBH-4 (Matilainen et al., 2013). In addition, it has been shown that graded depletion of RPN-10, a component of the 19S lid like RPN-6.1, results in an adaptive response that can compensate for compromised proteasome activity (Keith et al., 2016). Moreover, proteasomes can adapt to specific stress conditions (Yun et al., 2008) and show highly tissue-specific changes when different proteasomal subunits are depleted (Mikkonen et al., 2017). Collectively, this demonstrates that developmental stage- or cell-specific loss of presumably essential proteasome subunits does not lead to pleiotropic phenotypes, but in many cases reveals highly context-dependent aspects of ubiquitin proteasome system function.

We also demonstrate that head morphogenesis in general and dendrite morphogenesis in particular are an ideal developmental scenario to study *de novo* apicobasal polarization. *De novo* apical polarization has been characterized for cysts and during vasculogenesis (Bryant et al., 2010; Lenard et al., 2015), however, in these contexts, polarization strictly depends on concomitant lumen formation and is often directly coupled to mitotic dynamics (spindle and midbody position). In the case of sensory organ morphogenesis, mitotic cues are lacking since cells are already post-mitotic. Only a small fraction of cells, the socket glia cells, form a *bona fide* lumen as demonstrated by electron microscopy. Thus, all participating anterior located cells are non-polar at the start of the process as judged by the absence of the known apical PARs and non-muscle myosin II. Therefore, they need to polarize by different cues than those known so far. We would like to suggest that a developmental timer together with an extrinsic cue, the apical extracellular matrix (aECM), most likely organize collective apical polarization. Remarkably, a main component of the aECM is DEX-1, a secreted protein with nidogen and EGF-like domains which is essential for embryonic development (Cohen et al., 2019). DEX-1 is expressed in epithelial tissues that build the aECM during embryogenesis. Importantly, *dex-1* mutants have a pharynx ingressed (Pin) phenotype, implying that the pharynx lumen ends inside the embryo and more anteriorly positioned cells occlude it. In the light of our findings, a very likely interpretation of these data is that without a properly formed aECM, anteriorly located cells might not undergo apical constriction, which leads to sensory organ dendrite phenotypes (Heiman and Shaham, 2009). More importantly, it could lead to a lack of delamination of other apically polarizing cells and failed or partially failed mouth morphogenesis because this requires arcade cell apical constriction (Figure 8D). In addition, Cohen et al. (2019) were not able to detect a sperm protein zonadhesin homology that was previously proposed (Heiman and Shaham, 2009) and demonstrate that *dex-1* null mutants have more severe phenotypes than truncation mutants used previously. Therefore, we argue that the function of DEX-1 should be re-interpreted and re-investigated in the context of *de novo* apical polarization and collective morphogenesis. Notably, we can exclude that formation of the basement membrane around the pharynx serves as the cue for polarization since *de novo* apically polarizing cells are not directly contacting the pharynx primordium when overt apical polarization of anterior cells occurs. The data presented here strongly suggest that during dendrite morphogenesis, glia cells in *C. elegans* can be considered interstitial cells whose main function is to form the connection between epidermal tissues and neurons. We also present evidence that the arcade cells might fulfill a very similar function morphogenetically: they form the connection to the outside by forming an anterior pore, and, subsequently connect the lumen of the pharynx to this pore. This view is also supported by the fact that socket glia and arcade cells show a striking similarity in their shape at the end of embryonic development (Altun and Hall, 2009b).

Taken together, our data unveil two morphogenetic modules that organize the *C. elegans* sensory organs, dendrite towing of a pre-assembled sensory organ in the epidermis and apical constriction-mediated placement of sensory organs around the mouth opening. This supports the idea of co-evolution of epidermal patterning and neuro-morphogenesis so that gradually, simple neuroectodermal tissues—where a simple epithelium contains a few sensory neurons that form a sparse network—can be transformed into epithelial systems with complex topologies and fasciculated sensory organs (Holland, 2003).

## Data Availability Statement

The original contributions presented in the study are included in the article/Supplementary Material, further inquiries can be directed to the corresponding author/s.

## Author Contributions

CP conceived and supervised the project, and wrote the manuscript with input from all other authors. PK, CL, and CP performed the experiments. PK and CP analyzed the data. All authors contributed to the article and approved the submitted version.

## Conflict of Interest

The authors declare that the research was conducted in the absence of any commercial or financial relationships that could be construed as a potential conflict of interest.
