# Supplementary material for: Differential Thresholds of Proteasome Activation Reveal Two Separable Mechanisms of Sensory Organ Polarization in C. elegans
Source: Front Cell Dev Biol. 2021 Feb 9;9:619596. doi: 10.3389/fcell.2021.619596 (PMC7900421; doi:10.3389/fcell.2021.619596)
Supplement: Supplementary file 14 [file Data_Sheet_1.pdf]

Differential thresholds of proteasome activation reveal two separable mechanisms of sensory organ dendrite polarization in *C. elegans*

Patricia Kunz, Christina Lehmann, Christian Pohl

Supplementary Material

Strains used in this study

**Strain**	**Genotype**
BV43	(FT17xOD70); xnIs3[par-6:PAR-6::GFP + unc-119(+)]; ltIs44 [pie-1p-mCherry::PH (PLC1delta1) + unc-119(+)]V.
BV24	(OD70xRW10029); ltIs44 [pie-1p-mCherry::PH(PLC1delta1) + unc-119(+)]; zuIs178 [his-72(1kb 5' UTR)::his-72::SRPVAT::GFP::his-72 (1KB 3' UTR) + 5.7 kb XbaI - HindIII unc-119(+)]; stIs10024 [pie-1::H2B::GFP::pie-1 3' UTR + unc-119(+)].
CHP56	(ML916xBV43) mcIs40 [lin-26p::ABDvab-10::mCherry + myo-2p::GFP]; xnIs3 [par-6:PAR-6::GFP + unc-119(+)]
CHP71	(ML916xSX621); mcIs40 [lin-26p::ABDvab-10::mCherry + myo-2p::GFP]; lin-15B&lin-15A(n765) X; mjIs27; mjIs27 [mir-124p::GFP + lin-15(+)].
CHP200	(SX392xKK1248); mjEx142 [mir-124p::mCherry]; par-6(it319[par-6::GFP]) I.
SM481	pxIs10 [pha-4::GFP::CAAX + (pRF4) rol-6(su1006)].
BV36	(JJ1473xOD70); zuIs45[nmy-2::NMY-2::GFP + unc-119(+)]; ltIs44 [pie-1p-mCherry::PH(PLC1delta1) + unc-119(+)] V.
VH624	rhIs13 V; nre-1(hd20) lin-15B(hd126) X. rhIs13 [unc-119::GFP + dpy-20(+)].
BV113	zbIs2 [pie-1::lifeact-RFP + unc-119(+)]; zuIs45[nmy-2::NMY-2::GFP + unc-119(+)] IV.
BV34	(AZ235xOD70); ruIs48[unc-119(+) + pie-1::GFP::tubulin]; ltIs44 [pie-1p-mCherry::PH(PLC1delta1) + unc-119(+)] V.
CHP201	(JH2825xSX621); unc-119(ed3) III; axIs1943 [(pFM050) mCherry::mlc-4 + unc-119(+)]; lin-15B&lin-15A(n765) X; mjIs27 [mir-124p::GFP + lin-15(+)].
CHP19	(LP162xJH2647); nmy-2(cp13[nmy-2::GFP + LoxP]) I; axIs1928 [mCherry::par-6].
CHP13	(JH2825xKK1216) unc-119(ed3) III; axIs1943 [(pFM050) mCherry::mlc-4 + unc-119(+)]; par-3(it298[par-3::GFP]) III.

Supplementary Figures

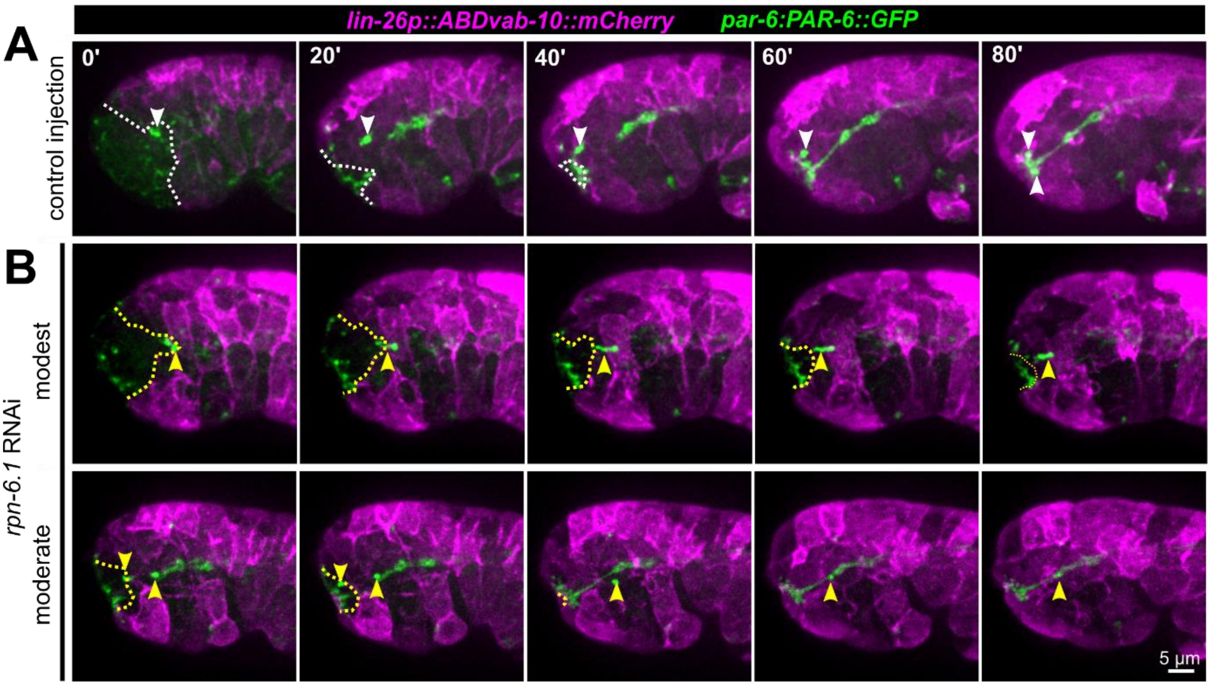

Supplementary Figure S1. Phenotypes of *rpn-6.1* RNAi embryos revealed by using polarity and epidermal markers. **A|** Maximum intensity projections of stacks of confocal images from time lapse recordings showing a left side view of a representative embryo (lima-bean to 1.5-fold stage) after control injection illustrating the movement of sensilla pores and the epidermal sheet. White dashed lines mark the anterior margin of the epidermal sheet. White arrowheads mark the AM sensillum pore. **B|** Depictions as in A, however, left/dorsal view of representative embryos after *rpn-6.1* dsRNA injection is shown. White arrowheads are pointing to the amphid pores undergoing wt morphogenesis. Yellow arrowheads highlight impaired amphid pore establishment or migration. Yellow dashed lines mark the margin of the epidermal sheet after *rpn-6.1* depletion. Yellow asterisks indicate pharynx to gut connection defects. Scale bar = 5 µm.

Supplementary Figure S2. Detailed analysis of *rpn-6.1* RNAi phenotypes.

(green)	pore ok	(lilac)	dendrites ok	(light green)	pharynx/intestine ok
(yellow)	pore impaired	(blue)	elongation impaired	(green)	no connection pharynx to mouth
(red)	pore arrests	(magenta)	no elongation	(green)	intestine morphogenesis impaired

n sensilla = 29 Embryos

n pharynx & intestine = 22 Embryos

Quantification of phenotypes after RPN-6.1 depletion

Embryo	pore left	dendrites left	pore right	dendrites right	pharynx	intestine
270819_sess2_E1	split but find back together, migration	ok	lost	no elongation	ok	interrupted
270819_sess2_E2	split , posterorerior part arrests	strong impaired elongation	long, partwise split, getting together	ok	ok	ok
270819_sess2_E3	long, migratrion ok	ok	ok	ok	ok	interrupted
270819_sess3_E1	nearly split, migration	ok	split, posterior part arrests	elongation till posterior part split pore, still partly working	no connection	ok
270819_sess3_E2	split, still migration	elongation till posterior part pore	ok	ok	ok	ok
270819_sess3_E4	split, posterior part arrests	most dendrites no elongation . Those attached to poeterior part pore, thin bundle reaches anterior part	lost	no elongation	no connection	no throughgut
270819_sess4_E1	split, posterior part migrates slowly	slow elongation	partwise split/long, gets back together, migration	ok	no connection	no throughgut
270819_sess4_E3	ok	ok	long, migrating ok	ok	no connection	not normal
270819_sess4_E4	partwise split, migrates	ok	ok	ok	no connection	strong interruptions
290819_sess1_E4	arrest	no elongation, messed up	no correct migration	no elongation, messed up	no connection	okish
290819_sess2_E1	partwise split, gets together, migration	ok	slower migration	ok	ok	ok
290819_sess2_E2	loong, part split?	ok	loong, part split?	ok	ok	ok
290819_sess2_E3	split, posterior part arrests	no elongation	split, posterior part arrests	no elongation, messed up		
290819_sess2_E5	loong, partwise split	elongation slower	think ok, not well visible	think ok, not visible well	ok	interrupted
290819_sess3_E1	loong, migration ok	ok	lost	no elongation		
290819_sess3_E3	partly split, migration ok	ok elongation till posterior part, thinner bundle till anterior part, elongation works	not visible well	not visible well	ok	ok
290819_sess3_E4	spatio-temporal failure, split? migration	no elongation	not visible well	not visible well		
100919_sess1_E2	loong, still migrating	ok	loong, migrating okish	ok	ok	ok
100919_sess1_E3	not properly established	no proper migration, just small bundle migrates	pore split, posterior part arrests	no elongation	no connection	no throughgut
100919_sess1_E4	in between split, migrating fine	ok	split, posterior part stuck, anterior part migration	elongation just till posterior part pore properly, very thin bundle till anterior pat	ok	ok
100919_sess1_E5	part pore stuck	no elongation	no pore?	no elongation	no connection	getting disrupted
100919_sess1_E6	just part pore? Migration	very poor elongation, very thin bundle	pore migrating, or just part pore?	very poor elongation, thin bundle		
100919_sess2_E1	loong, migrating	elongation happening impaired	not visible	not visible		
100919_sess2_E2	pore long, partly falling apart, stil migrating	ok	not visible	not visible	ok	ok
100919_sess2_E3	partwise long, migration	ok	not vsible	not visible		
100919_sess2_E4	migration ok	okish	not vsible	not visible	no connection	no throughgut
100919_sess3_E1	pore split no properl migration	elongation till posterior part pore	split, posterior part arrests	no proper elongation, just small bundle	no connection	no throughgut, but gut tissue dying?
100919_sess3_E3	pore split, tiny part migration	elongation thin bundle?	ok	ok		
100919_sess3_E4	pore very split, some migration	elongation happens impaired	not visible	not visible	ok	ok, shape not proper

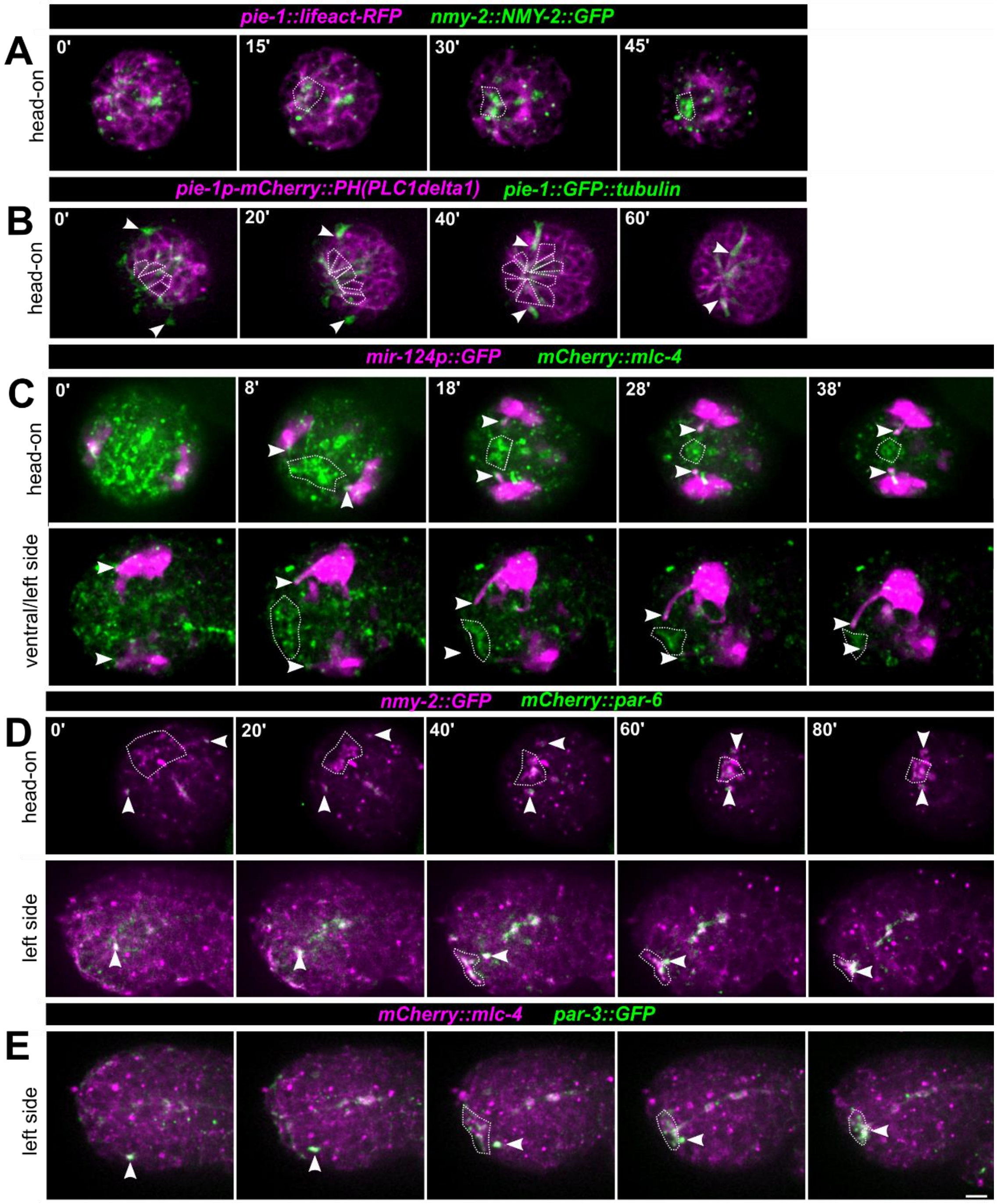

Supplementary Figure S3. Additional markers collective apical constriction. **A|** Head-on view of a wt embryo between lima-bean and 1.5-fold stage illustrating the accumulation of NMY-2 (green, NMY-2) together with cell shape changes (plama membranes, magenta). **B|** Same representation as in A, however, showing tubulin (green) as a marker for bundled dendrites (their tips marked by arrowheads). Anterior-most cells are outlined. **C|** Head-on (top) and ventral/left side views of wt embryos expressing myosin ligh chain (green, MLC-4) and an AM marker (magenta, *Pmir-124*). Anterior accumulation of MLC-4 is outlined and dendrite tips are marked by arrowheads. **D|** Same depictions as in C, however, embryos express NMY-2 (magenta) and PAR-6 (green). **E|** Same depictions as in the lower panel of D, however, embryos express MLC-4 (magenta) and PAR-3 (green). All fluorescence images are maximum intensity projections of stacks or single layer confocal images from time lapse recordings. Scale bar = 5 µm.

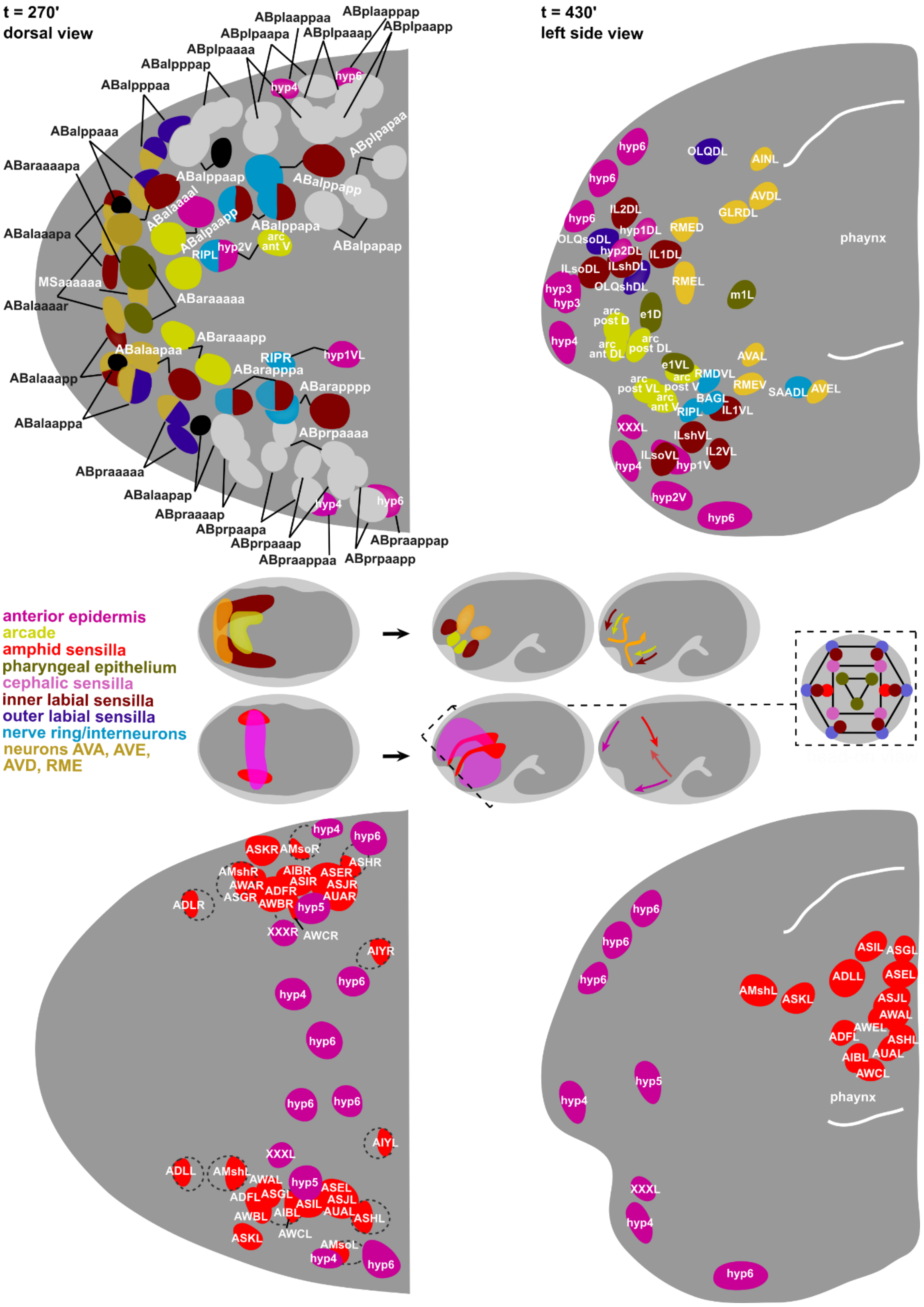

Supplementary Figure S4. Annotated map of cell-cell re-arrangements. Top and bottom panels: Renderings of cellular positions at 270 min (left) and 430 min (right) of embryonic development drawn after the maps shown in Sulston et al., 1983. Colors indicate final cell type (see legend in the middle panel). Middle panel: Cellular re-arrangements that need to result from the cellular maps. See main text for details.

Supplementary Videos

Supplementary Video S1: AM pore movement

Representative time lapse series showing PAR-6 as a marker for sensory organ pore movement (AM pores (white arrowheads) in wt lima bean to 1.5-fold stage embryos). The left panel shows a dorsal/left side view and the right panel an anterior view. White circles highlight deirid pores (anterior), excretory pore (ventral) or phasmids (posterior). Additionally, a white line is encircling the intestine. Scale bar = 5 µm.

Supplementary Video S2: AM cell lineage tracing

Representative time lapse series using HIS-72 marker for lineage tracing of AMsoL, AMshL, ASEL, XXXL and hyp5 (left) cells from 4-cell stage until 2-fold stage embryo. Shown is the left side of the wt embryo. Scale bar represents 5 µm.

Supplementary Video S3: AM pore & epidermis

Representative time lapse series illustrating the wt early (top panels) and later (bottom panels) head epiboly (white line) and the translocation of the AM pores (white arrowheads) using PAR-6 and VAB-10 markers. Left panels show lateral view while right panels show head-on view of early lima bean to 1.5-fold stage embryos. Scale bar represents 5 µm.

Supplementary Video S4: UV Laser ablation of epidermis

Top panel shows wt AM pore translocation (white arrowhead). Two representative embryos (middle and bottom right panels) are illustrating the AM pore arresting (yellow arrowheads) after UV laser ablation of the epidermis close to one pore each (middle and bottom left panels, white circles). The pores on the other side of the same embryo move like in wt (white arrowheads). Left side view of all embryos. Scale bar represents 5 µm.

Supplementary Video S5: AM pore movement, AM dendrite elongation

Representative wt embryos from early lima bean to 1.5-fold stage illustrating the AM pore translocation (white arrowheads) by PAR-6 marker and AM dendrite extension highlighted through *pmir-124* marker. Movement of additional sensilla pores is emphasized by a white line. Phasmids are highlighted by a blue arrowhead. Left panel shows dorsal/left side view and right panel head-on view. Scale bar represents 5 µm.

Supplementary Video S6: AM dendrites and epidermal morphogenesis

Representative time lapse series showing wt epidermal ventral enclosure and early head epiboly (top panel, white line) and later head epiboly (bottom panels, white line), the latter collectively with the AM

dendrite elongation (white arrowheads). Epidermis is marked by VAB-10 and AM dendrites by *pmir-124* marker. Top panel shows ventral side, bottom left panel lateral and bottom right panel head-on view of early lima-bean to 1.5-fold stage embryos. Scale bar represents 5 µm.

Supplementary Video S7: UV Laser ablation of AM pore

Representative wt embryos from lima bean to 1.5-fold stage before (left panel, ablation position white circle) and after AM pore laser ablation (middle panel) compared to wt (right panel, AM pores white arrowheads). The ablated side is showing impaired AM dendrite elongation (yellow arrowhead) while the not ablated pore on the other side of the same embryo shows wt translocation (white arrowhead). AM sensilla on ablated side are still forming intact commissures (blue arrowhead). AM pores are marked by PAR-6 and AM dendrites by *pmir-124*. Ventral up/left side view. Scale bar represents 5 µm.

Supplementary Video S8: RPN-6.1 depletion-induced AM pore and dendrite phenotypes

Representative embryos (lima bean to 1.5-fold stage) after RPN-6.1 depletion showing gradually impaired AM pore (PAR-6) movement and AM dendrite extension (second top to bottom panels, yellow arrowheads, *pmir-124*) compared to control injection (white arrowheads). Even when AM pore translocation is strongly impaired (middle panel), or the AM pore and dendrite extension arrest (bottom panel), all other sensilla pores are reaching the anterior tip of the embryo (white line). Ventral up/left side view. Scale bar represents 5 µm.

Supplementary Video S9: RPN-6.1 depletion induced intestinal phenotypes

Representative embryos (lima bean to 1.5-fold stage) after RPN-6.1 depletion showing gradually impaired pharynx and intestine development (second top to bottom panels, yellow asterisks/arrowheads) compared to control injection (top panel). PAR-6 is used to mark the pharynx and intestine. Dorsal up/left side view. Scale bar represents 5 µm.

Supplementary Video S10: Apical constriction reflected by cell shape changes

Representative wt embryo (lima bean to 2-fold stage) highlighting anterior, apically constricting cells (outlined in white). NMY-2 expression accumulating at the prospective mouth is outlined in blue. Ventral up view. Markers are NMY-2 and *Ppie-1-PH(PLCd1)* (plasma membranes). Scale bar represents 5 µm.

Supplementary Video S11: Accumulation of apical polarity components during neurite elongation

Representative embryos (lima bean to 1.5-fold stage) highlighting the accumulation of non-muscle-myosin components MLC-4 (middle panel) or NMY-2 (bottom panel) and tubulin (top panel), head-on views. Tubulin bundles are translocating anteriorly near to the prospective mouth (top panel, green); both MLC-4 (green, middle panel) and NMY-2 (magenta, bottom panel) are enriched at the position of the prospective mouth (middle and bottom panels, white lines). Elongation of the AM neurites (top and

middle panel) or the migration of the pores (bottom panel) are highlighted by arrowheads. Scale bar represents 5 µm.

Supplementary Video S12: RPN-6.1 depletion and collective shape change phenotypes

Representative embryos (lima bean to 1.5-fold stage) demonstrating first bottle-shaped cells at the anterior-most tip (top panel, white outlines) and the translocating AM pore (white arrowhead) after control injection. RPN-6.1 depletion leads to loss of cell shape changes (bottom panel, yellow outlines) and AM pore translocation arrests (yellow arrowheads), however, sensilla pores still migrate to the prospective mouth (PAR-6 signals, green). Left side view using PAR-6 as sensilla pore marker and highlighting the membrane through *Ppie-1-PH(PLCd1)*. Scale bar represents 5 µm.

Supplementary Video S13: Arcade cell shape changes

Representative wt embryo (lima bean to 2-fold stage) expressing *Ppha-4::GFP::CAAX,* a *Ppha-4*-driven prenylated GFP with arcade cells (red outlines), pharynx (green outlines) and gut (blue outlines) highlighted is shown from ventral (start) turning sideways during elongation. Note that towards the end of the movie, arcade expression of the marker decreases and the apically constricted anterior pharynx cells becomes prevalent. Scale bar represents 5 µm.

Supplementary statistical analysis details

Two-way ANOVA (mixed-effects model) for Figure 4D:

AM pore – distance to mouth

Table Analyzed	pore abl_pore			
**Mixed-effects model (REML)**	Matching: Stacked			
Assume sphericity?	No			
Alpha	0.05			
**Fixed effects (type III)**	**P value**	**P value summary**	**Statistically significant (P < 0.05)?**	**F (DFn, DFd)**
Row Factor	0.1160	ns	No	F (0.02386, 0.1591) = 29.22
Column Factor	0.0861	ns	No	F (1, 8) = 3.829
Row Factor x Column Factor	0.0037	**	Yes	F (6, 40) = 3.901
**Random effects**	**SD**	**Variance**		
Subject	0.04754	0.002260		
Residual	0.02010	0.0004038		
**Was the matching effective?**				
Chi-square, df	65.88, 1			
P value	<0.0001			
P value summary	****			
Is there significant matching (P < 0.05)'	Yes			
**Difference between column means**				
Predicted mean of not ablated side	0.1091			
Predicted mean of ablated side	0.1689			
Difference between predicted means	-0.05981			
SE of difference	0.03056			
95% CI of difference	-0.1303 to 0.01067			

AM dendrite extension – length

Table Analyzed	pore abl_dendrites			
**Mixed-effects model (REML)**	Matching: Stacked			
Assume sphericity?	No			
Alpha	0.05			
**Fixed effects (type III)**	**P value**	**P value summary**	**Statistically significant (P < 0.05)?**	**F (DFn, DFd)**
Row Factor	0.0005	***	Yes	F (2.552, 17.01) = 10.69
Column Factor	0.0140	*	Yes	F (1, 8) = 9.809
Row Factor x Column Factor	0.0004	***	Yes	F (6, 40) = 5.309
**Random effects**	**SD**	**Variance**		
Subject	0.04109	0.001688		
Residual	0.02052	0.0004209		
**Was the matching effective?**				
Chi-square, df	51.71, 1			
P value	<0.0001			
P value summary	****			
Is there significant matching (P < 0.05)'	Yes			
**Difference between column means**				
Predicted mean of not ablated side	0.1454			
Predicted mean of ablated side	0.06214			
Difference between predicted means	0.08325			
SE of difference	0.02658			
95% CI of difference	0.02195 to 0.1446			

Two-way ANOVA (mixed-effects model) for Figure 5C:

AM pore – distance to mouth

Table Analyzed	pore_rpn-6.1			
**Mixed-effects model (REML)**	Matching: Stacked			
Assume sphericity?	Yes			
Alpha	0.05			
**Fixed effects (type III)**	**P value**	**P value summary**	**Statistically significant (P < 0.05)?**	**F (DFn, DFd)**
Row Factor	<0.0001	****	Yes	F (5, 28) = 55.37
Column Factor	0.0002	***	Yes	F (1, 7) = 53.54
Row Factor x Column Factor	<0.0001	****	Yes	F (5, 28) = 18.40
**Random effects**	**SD**	**Variance**		
Subject	0.03255	0.001059		
Residual	0.02056	0.0004225		
**Was the matching effective?**				
Chi-square, df	22.15, 1			
P value	<0.0001			
P value summary	****			
Is there significant matching (P < 0.05)?	Yes			
**Difference between column means**				
Predicted mean of RPN-6.1 depletion pore	0.2623			
Predicted mean of control pore	0.09559			
Difference between predicted means	0.1667			
SE of difference	0.02278			
95% CI of difference	0.1128 to 0.2205			

AM dendrite extension – length

Table Analyzed	dendrite_rpn-6.1			
**Mixed-effects model (REML)**	Matching: Stacked			
Assume sphericity?	No			
Alpha	0.05			
**Fixed effects (type III)**	**P value**	**P value summary**	**Statistically significant (P < 0.05)?**	**F (DFn, DFd)**
Row Factor	0.0035	**	Yes	F (0.9757, 5.464) = 24.18
Column Factor	0.0370	*	Yes	F (1, 7) = 6.604
Row Factor x Column Factor	0.0002	***	Yes	F (5, 28) = 7.109
**Random effects**	**SD**	**Variance**		
Subject	0.03840	0.001475		
Residual	0.02357	0.0005557		
**Was the matching effective?**				
Chi-square, df	28.50, 1			
P value	<0.0001			
P value summary	****			
Is there significant matching (P < 0.05)?	Yes			
**Difference between column means**				
Predicted mean of RPN-6.1 depletion dendrit	0.04546			
Predicted mean of control dendrite	0.1144			
Difference between predicted means	-0.06890			
SE of difference	0.02681			
95% CI of difference	-0.1323 to -0.005501			
